# Immunogenic mapping of rDyn-1 and rKDDR-plus proteins and selection of oligopeptides by immunoblotting for the diagnosis of *Leishmania infantum*-infected dogs

**DOI:** 10.1371/journal.pntd.0011535

**Published:** 2023-08-04

**Authors:** Williane Fernanda Siqueira, Mariana Santos Cardoso, Vanessa Gomes Fraga, Jennifer Ottino, Vitor Márcio Ribeiro, Carolina Novato Gondim, Joziana Muniz de Paiva Barçante, Ana Carolina Amado Gomes, Alexsandro Sobreira Galdino, Kasper Eersels, Bart van Grinsven, Daniella Castanheira Bartholomeu, Lilian Lacerda Bueno, Thomas Cleij, Ricardo Toshio Fujiwara

**Affiliations:** 1 Department of Parasitology, Universidade Federal de Minas Gerais, Belo Horizonte, Brazil; 2 Faculty of Science and Engineering of Maastricht University, Maastricht, Limburg, Netherlands; 3 Veterinary School, Pontificial Catholic University of Minas Gerais, Betim, Brazil; 4 Department of Health Sciences, Universidade Federal de Lavras, Lavras (MG), Brazil; 5 Laboratory of Microbial Biotechnology, Universidade Federal de São João Del-Rei, Divinópolis, Minas Gerais, Brazil; Institut Pasteur de Tunis, TUNISIA

## Abstract

Endemic in Brazil, visceral leishmaniasis (VL) is a zoonotic infection that is among the most important parasitic diseases transmitted by vectors. Dogs are the main reservoirs of canine leishmaniasis (CanL) and their identification is used in some countries as part of disease prevention and control measures in the canine and human population. In this context, serological tests are necessary, composed of antigens capable of correctly identifying infected dogs, minimizing the number of false-negative cases. This study aimed to identify more immunoreactive peptides derived from two previously described whole proteins (rDyn-1 and rKDDR-plus) and compare their performance to the control antigens rK39 and the crude extract for the detection of dogs infected with *L*. *infantum*, especially the asymptomatic ones. The three selected peptides and a mixture of them, along with the rDyn-1, rKDDR-plus, rK39, and crude extract antigens were evaluated using indirect ELISA with sera samples from 186 dogs with CanL, being asymptomatic (n = 50), symptomatic (n = 50), co-infected (n = 19), infected with *Babesia* sp. (n = 7), *Ehrlichia* sp. (n = 6), *T*. *cruzi* (n = 20) and uninfected (n = 34). The results showed that the rDyn-1 protein and the peptide mixture had the highest sensitivity (100% and 98.32%, respectively) and specificity (97.01 and 98.51, respectively). A high degree of kappa agreement was found for rDyn-1 protein (0.977), mixed peptides (0.965), rKDDR-plus protein (0.953), K-plus peptide 1 (0.930) and Dyn-1 peptide (0.893). The mixture of peptides showed the highest likelihood (65.87). The ELISA using the mixture of peptides and the rDyn-1 protein showed high performance for CanL serodiagnosis. More mix combinations of the peptides and additional extended field tests with a larger sample size are recommended.

## Author summary

Canine leishmaniasis (CanL) is a parasitic zoonotic infection of dogs caused by *Leishmania* spp. parasites. In Brazil, CanL is among the most important vector-borne parasitic diseases. Dogs are the main reservoirs of the disease and in some countries have been regularly culled as part of government policy to control human leishmaniasis. The identification of infected dogs, regardless of the clinical condition, is necessary as part of the prevention and control measures of the disease in the canine population and consequently in the human population. In this context, it is necessary serological tests, composed of antigens capable of correctly identifying infected, asymptomatic, as well as symptomatic dogs, minimizing the number of false negative cases. Therefore, this study aimed to compare the efficacy between two previously described whole proteins (rDyn-1 and rKDDR-plus) with their more immunoreactive synthetic peptides to sera from symptomatic and asymptomatic dogs selected in immunoblotting assays and to evaluate in ELISA assays. ELISA using the rDyn-1 protein and mixed peptides showed high performance in the diagnosis of CanL.

## Introduction

Visceral leishmaniasis (VL), also known as Kala-azar, is a serious disease with a wide spectrum of clinical manifestations, which can lead to the death of the individual if not diagnosed and treated early. The VL is on the list of neglected tropical diseases and is considered a disease of high impact on public health, affecting thousands of dogs and humans annually [1]. This disease is responsible for about 50,000 to 90,000 new cases annually, of which only 25 to 45% are reported to the WHO (World Health Organization) [2]. Ten countries are responsible for more than 90% of VL cases in the world, including Brazil, China, Ethiopia and India [3].

In the Americas, VL is a zoonosis caused by the obligate intracellular protozoan parasite *L*. *infantum*, and domestic dogs (*Canis familiaris*) are considered the main source of infection [4]. The *L*. *infantum* infection in dogs named canine leishmaniasis (CanL) is a multi-systemic disease with a range of non-specific clinical signs that when present can vary from lymphadenomegaly, splenomegaly, cutaneous and ocular lesions to nephropathies [5–7]. There is a direct association between the number of cases of human and canine disease because, despite the data indicating a higher prevalence of the disease in dogs than in humans, the presence of infected animals contributes to the increased risk of human disease [8]. Therefore, the appearance of cases of VL in humans is preceded by canine enzootic.

Animals with CanL are mostly asymptomatic, about 80%, and may not be correctly diagnosed by serological tests because they have a low, fluctuating, or even absent antibody response to the parasite antigens or because they are in the initial phase of the disease [9,10]. Asymptomatic dogs or dogs with non-apparent infections are those in which there is no evidence of clinical manifestations or absence of clinical signs suggestive of *Leishmania* infection residing or staying for a long time in areas considered endemic for leishmaniasis, and that present positive diagnostic in a combination of tests (serological, molecular, and/or parasitological) [11]. Although the clinical disease do not be apparent in these animals, they are bearers of *Leishmania*, being, therefore, an important source of disease reservoirs, being as capable of transmitting the parasite to the vector as well as the symptomatic dogs. [12–14]. Therefore, tests capable of diagnosing early infections in dogs with leishmaniasis, especially asymptomatic dogs, has a significant role in the management and control of human and canine disease.

Parasitological methods, based on direct microscopic visualization or culture of the parasite, are considered the gold standard for diagnosing the disease, as they have 100% specificity [15]. However, their sensitivity is highly variable, they are dependent on biological material from aspiration biopsy of the spleen, bone marrow or lymph nodes, making them highly invasive and risky, in addition to not having applicability in the field [16]. In turn, serological methods appear as an alternative to invasive parasitological methods, since VL is characterized by a large production of specific antibodies [15]. A vast number of serological techniques are available for the diagnosis of the disease, allowing a wide spectrum of sensitivity and specificity, excellent cost-effectiveness and applicability in the field and in mass. [17]. Indirect Fluorescent Antibody Test (IFAT), Enzyme Linked Immunosorbent Assay (ELISA), Direct Agglutination Test (DAT), Immunochromatographic (ICT) strip are serological tests most used in the diagnosis of the disease. ELISA and ICT are simple, accurate and efficient serological tests whose performance is mainly related to the type of antigen used. The use of crude extract in this technique has been shown to be a limiting factor of its specificity, in this context, recombinant proteins appear as an alternative. In addition to improving diagnostic accuracy, recombinant proteins allow large-scale production in a standardized way and independent of *Leishmania* growth in laboratory cultures. [18].

We have recently demonstrated the potential of two recombinant antigens (Dyn-1 and KDDR-plus), derived from *L*. *infantum* for the diagnosis of CanL belonging to two protein superfamilies dynamin-1-like and kinesin, respectively. The protein rDyn-1 showed an excellent ability to identify asymptomatic dogs [19]. While that, the protein rKDDR-plus presented excellent for discriminating dogs that have the infection regardless of the clinical status of the animal, presenting low cross-reaction with other organisms [20]. Both the dynamin-1-like and kinesin protein superfamily are involved in several cellular processes essential for cells reflecting in dynamic properties, similar to molecular motors [21,22]. Besides the rKDDR-plus other Kinesin-related conserved recombinant antigens have already been reported in the literature as potential targets for the diagnosis of CanL. In this scenario, stand out the antigens rK39 [23], rKDDR [24], rK9 [25], rK26 [25] rK28 [21], rKRP42 [26], rKE16 [27] and rKLO8 [28]. Already antigens derived from dynamins were linked to the diagnosis of CanL for the first time in the work of Siquiera et al [19].

In view of the promising results obtained by both proteins, it was thought in to identify peptides present in the rDyn-1 protein, responsible for the ability to identify the asymptomatic dogs, as well as the symptomatic ones. In addition to identifying, the peptides present in the rKDDR-plus protein responsible for the ability to discriminate dogs that have *L*. *infantum* infection maintaining a low cross-reactivity index with other organisms. Therefore this study aimed to identify peptide sequences present in two complete proteins, rDyn-1, and rKDDR-plus, and to select potential immunoreactive candidates to sera from dogs with *Leishmania* to be used in the serodiagnosis of dogs with CanL, mainly in asymptomatic dogs.

## Methods

### Ethics statement

For the selection of immunoreactive peptides in the immunoblotting assays, previously characterized canine sera were used. All collection procedures and anesthetic procedures were performed exclusively by a veterinarian, in accordance with art. 3, of Resolution N° 877, of February 15, 2008, of the CFMV. The clinical data and samples from dogs with CanL, as well as the permission for data use, were provided by the Ethics Committee on Animal Use of the Federal University of Minas Gerais, Brazil (CEUA) under protocol number 44/2012.

### Serum samples

For this study were used a total of 186 serum samples were from male and female dogs of various breeds and ages belonging to a shelter or not. Veterinarians with the consent of the owners or shelter representatives obtained biological samples and performed a physical examination of each animal. Dogs with positive molecular and serological tests for *L*. *infantum* were included in the group of dogs with CanL, this group in turn was subdivided into two other groups. Dogs with any suggestive sign of the disease such as skin changes (alopecia, furfuraceous eczema, ulcers, hyperkeratosis), hepatomegaly, hyperthermia, lymphadenopathy, splenomegaly, onychogryphosis, weight loss, keratoconjunctivitis, and hindlimb paresis without direct relation with any other disease were characterized as symptomatic [29]. On the other hand, the absence of clinical signs suggestive of *Leishmania* infection and that present positive diagnostic in a combination of tests (serological, molecular, and/or parasitological) was used to define dogs with asymptomatic clinical status.

Sera from 100 dogs (50 symptomatic and 50 asymptomatic), naturally infected, from endemic areas of Brazil (Montes Claros, Minas Gerais State, Brazil) to CanL and with the presence of the disease vector were included. In addition, 19 dogs co-infected with *Babesia* sp. and *L*. *infantum*. All this samples were tested by enzyme-linked immunosorbent (ELISA EIE) and immunochromatographic (Dual Path Platform: DPP) both from Biomanguinhos (official protocols recommended by the Brazilian Ministry of Health–screening by DPP and confirmation of reactive samples by ELISA) [30]. The positive molecular diagnoses were included by DNA extractions and real-time PCR method to detect the presence of *Leishmania* kDNA with positive serological and.

Samples from 33 seronegative dogs for anti-*Leishmania* antibodies (ELISA and rapid tests), but naturally or experimentally infected and with a positive diagnosis for other agents or affected by other confirmed pathological conditions were used to evaluate possible cross-reactions. This group was composed of naturally infected with *Babesia* sp. (n = 7) and *Ehrlichia* sp. (n = 6) and dogs experimentally infected with *T*. *cruzi* (n = 20). Samples from dogs naturally infected with *Babesia* sp. and *Ehrlichia* sp. were kindly provided from a private veterinary laboratory (Contagem/Minas Gerais State, Brazil). To confirm the positivity of the samples with *Babesia* sp. and *Ehrlichia* sp. the research for these infectious agents was carried out in blood smears in addition to real-time PCR molecular research using specific primers in peripheral and/or medullary blood to both parasites Serum samples infected with *T*. *cruzi* were kindly provided by the Department of Clinical Analysis of the School of Pharmacy/UFOP. The dogs were inoculated with 2.0×10^3^ bloodstream trypomastigotes per kg of body weight belonging to two strains; Y strain (DTU TcII), isolated from an acute human case of Chagas disease, and the Berenice-78 (Be-78) strain (DTU TcII) isolated by xenodiagnosis of a patient with an indeterminate form of the disease. The positivity to *T*. *cruzi* was confirmed by hemoculture or by combined positivity indicated by the Chagatest-ELISA Recombinante version 3.0 kit (Wiener Laboratorios, Santa Fé, Argentina) and Chagatest Indirect Hemagglutination Assay (IHA; Wiener Laboratorios).

Sera from 34 clinically healthy dogs seronegative by the tests recommended by the Brazilian Ministry of Health (ELISA assays and DPP) from areas where CanL is regarded as non-endemic was also included.

### Selection of the peptides derived from rDyn-1 and rKDDR-plus from spot synthesis

A bioinformatics prediction along the complete amino acid sequence of the rDyn-1 protein to identify the potentially immunoreactive peptide sequences was performed. rDyn-1 was subjected to B cell epitope predictions using the BepiPred 2.0 program (http://www.cbs.dtu.dk/services/BepiPred-2.0) with a cut-off of 0.6 [31]. Protein structural disorder prediction was performed using the IUPred program (http://iupred.elte.hu/), with a cut-off of 0.5 [32]. For the selection of peptides derived from the rKDDR-plus protein, other analyzes were performed.

The rKDDR-plus protein is characterized by the presence of 15.3 blocks of a sequence of 39 amino acids that are mostly repeated. However, these blocks present some degenerations, always varying between two amino acids in some sites. Therefore, in order to choose the peptides that were synthesized in the membrane, a vertical alignment of the 15.3 repetitive blocks was performed, the degenerate sites were identified and the amino acids that were most repeated in these sites were defined to compose the template sequence of 39 amino acids for the following analyses. Then, the 39 amino acids of the template sequence were fractionated into 10 amino acids with a sliding window of 2 amino acids and thus a simple mapping of the complete protein block for the choice of epitopes was performed. Both the peptides derived from the rDyn-1 protein, identified by bioinformatics predictions and the peptides derived from the rKDDR-plus protein, obtained in the scan, were subsequently synthesized in cellulose membranes.

### Spot synthesis in cellulose membrane

The peptide sequences, derived from the complete rDyn-1 and rKDDR-plus proteins were synthesized in a cellulose membrane, using an automatic synthesizer ResPep SL (Intavis) and the program MultiPep (Intavis), according to the SPOT synthesis technique [33,34]. Briefly, pre-activated and derivatized cellulose membranes were used for the multiple synthesis of peptide sequences in each spot in an automatic peptide synthesizer (Abimed Spot Synthesis–ASP222, Langenfeld, Germany) following the amino acid distribution plan, as well as the determination of the protocols of the various peptides defined in a computer program. Free hydroxyl groups on the cellulose membrane serve as an anchor point for peptide synthesis. These groups are coupled through a stable connection with 8 to 10 ethylene glycol units (Intavis AG), with the aim of moving the peptide away from the support and providing better stability in the connection of the peptide to the membrane. Peptide synthesis always starts at the C-terminus of the last amino acid of the established sequence. Free hydroxyl groups on the cellulose membrane serve as an anchor point for peptide synthesis. The amino acids are then activated by DIC (diisopropylcarbodiimide) Oxyma Pure at 1.1 M and deposited on the membrane in two coupling cycles without a new deprotection step between them. Free or unreacted NH_2_ functions are acetylated (3% acetic anhydride in DMF) in order to avoid side reactions with subsequently added amino acids or other undesirable bonds. The newly coupled Fmoc protecting group of the amino acid was again eliminated in the basic medium by piperidine at 25% v/v 4-methylpiperidine in DMF. A new amino acid dispensing cycle was then performed. These steps are repeated until the last amino acid is incorporated into the peptide being synthesized. At the end of the synthesis, the amino acid side groups are deprotected by the addition of 95% (v/v) trifluoroacetic acid (TFA) associated with 2.5% v/v water and 2.5% (v/v) triisopropylsilane (TIPS) to remove the protecting groups from the amino acid side chains, and thus the peptides remain covalently attached to the membrane. After the membrane was washed 4 times with dichloromethane (DCM), 4 times with DMF, and 2 times with ethanol. The membrane was dried, and the stains were checked under ultraviolet light. The membrane with the various identified peptides was subsequently analyzed by immunoblotting immunoassays using pools of canine sera.

### Screening of SPOT membranes

An immunological screening, by means of immunoblotting assays, was carried out to evaluate the serological performance of the peptides synthesized in the cellulose membrane. Initially, the membranes were incubated with pools of sera considered controls, with the first incubation performed with the pool of sera from dogs not infected with *L*. *infantum* (healthy control), followed by the pool of sera from dogs infected with other parasites; as *T*. *cruzi*, then with the pool of *Babesia* sp., and finally the pool of dogs infected with *Ehrlichia* sp. After incubations with pools of control sera, incubations were performed with pools positive for *L*. *infantum*, first with the asymptomatic group and lastly with the symptomatic group. Each pool was used in immunoblotting assays separately and each membrane was incubated with the respective pools individually and on different days. For this purpose, the membrane was blocked with a PBS solution containing 5% BSA and 4% sucrose for 12–16 h under agitation. Then, it was washed 3 times with the washing solution (PBS + 0.1% Tween20) for 10 minutes and incubated for two hours with a pool of specific serum diluted in the proportion 1:500 in the washing solution (PBS + 0.1% Tween20). After incubation, 3 more 10-minute washes were performed with the washing solution, followed by incubation for 1 h with peroxidase-conjugated anti-dog IgG secondary antibody (Sigma-Aldrich), diluted 1:10000. The membrane was again washed 3 times with the washing solution and finally revealed by chemiluminescence, through the addition of Luminata Forte Western HRP substrate (Merck), using the ImageQuant LAS 4000 digital imaging system (GE Healthcare). After development, the membranes were regenerated (removal of all antibodies bound to it) so that it could be used again with another pool of sera. For this, the membranes were initially washed 3 times using NN’Dimethylformamide (DMF) for 10 minutes, followed by incubation for 12 to 16 hours with a denaturing solution (8M urea + 1% (v/v) SDS in milli-Q water) and more two washes with the denaturing solution for 30 minutes each, followed by three washes with an acid solution (55% (v/v) ethanol, 35% (v/v) milli-Q water, 10% (v/v) glacial acetic acid). Finally, the membrane was washed for 2 minutes with deionized water, followed by 2 washes of 5 minutes with ethanol to remove moisture, dried at room temperature, and stored dry at 4°C or promptly reused. It is also worth mentioning that each membrane can be reused about 30 to 40 times with different sera, allowing the identification of reactive peptides [33].

### Scanning and measurement of spot signal intensities

Densitometric analyzes were performed for a better visual comparison, restoring the staining pattern of the spots, normalizing the colors for the stain with the highest value in all membranes, and allowing a real comparison between them. For that, ImageJ software and the Protein Array Analyzer plugin (http://image.bio.methods.free.fr/ImageJ/?Protein-Array-Analyzer-for-ImageJ.html) were used. The densitometry values of each stain were calculated using color intensity. Thus, it was possible to characterize the reactivity of the peptides by comparing the five types of sera tested. In this case, as the color progresses on the scale, the greater the reactivity of that peptide. After densitometry analysis, the next step was to start the process of selecting peptides based on their specificity and reactivity. For this, some filtering and selection criteria were assigned to obtain potential targets. Criterion 1: the background color of the membrane was removed, that is, the four least reactive spots were selected, and the average was calculated followed by the subtraction of this value in each spot by this value. Criterion 2: exclusion of peptides that were reactive in the incubation with the pool of negative control sera, that is, peptides above the established cut-off (negative mean + 2x the standard deviation). Criterion 3: exclusion of poorly reactive peptides in the incubation with the pool of sera with *Leishmania*, that is, peptides with positive spots smaller than the cut-off (mean of the negatives + 3x the standard deviation). These selection criteria allowed an initial screening to eliminate the peptides of lesser potential for future analysis. Then, the remaining peptides were again evaluated to select only the spots recognized with high affinity only by the pool of sera from dogs with CanL (asymptomatic and symptomatic). In addition, having low or no recognition with pools of non-infected canine sera (negative control), that is, serum from dogs healthy dogs and sera from dogs infected with *T*. *cruzi*, *Babesia* sp. and *Ehrlichia* sp.

### Peptide synthesis

The selected peptides were synthesized in a solid phase, that is, in a soluble form by the method developed by Merrifield (1969) in a 25 μmol scale, using the automatic synthesizer ResPep SL (Intavis1) [35]. This synthesis method consists of fixing the C-terminal amino acid of the peptide on insoluble solid support and then extending the peptide chain by successive additions of residues from the C-terminal to the N-terminal portion. These amino acids have the amine group protected by the Fmoc group and their side chain is also protected by a protective group to avoid unwanted reactions. The insoluble solid support is normally a resin which is also protected by Fmoc. Rink Amide resin (Intavis) was used as a solid support. The protocol used for peptide formation is similar to the one used for synthesis in cellulose membranes. After the end of the last synthesis cycle, the peptide without the Fmoc group of the last amino acid was removed from the resin by a step called cleavage. In this step, the protective groups of the side chain are also removed. The synthesized peptides were solubilized, precipitated with cold methyl tert-butyl ether, and lyophilized. Then, the synthesized peptides went through production quality control to ensure that the peptides were synthesized correctly.

### Confirmation of peptide identity by mass spectrometry (MALDI/TOF)

The synthesized peptides were submitted to the MALDI method (Matrix Assisted Laser Desorption/Ionization) that allows the ionization of several macromolecules combined with the TOF/MS/MS system (Time of Fligh/Mass Spectrometer) (Bruker Daltonics) a kind of spectrometer of mass that utilizes the time-of-flight difference due to the size differences of the ionized substances.

For the analysis, 0.5 μL of the concentrated sample was mixed with 0.25 mL of a saturated matrix solution 10 mg/mL α-cyano-4-hydroxycinnamic (Aldrich, Milwaukee, WI) in 50% acetonitrile/0.1% trifluoroacetic acid. The samples were applied to an MTP AnchorChip 600/384 plate (Bruker Daltonics) and left to dry at room temperature. The raw data was obtained by MALDI-TOF/TOF Autoflex III (Bruker Daltonics, Billerica, USA) using a positive/reflector mode controlled by FlexControl 3.3 software. The instrument calibration was performed using reference peptides (Peptide Standard, Bruker Daltonics). Each spectrum was produced by accumulating data from 200 consecutive laser shots.

After confirming the identity of the synthetic peptides, they were subjected to serological ELISA assays using the same pools of sera previously used in the immunoblotting assays, in order to verify whether the results obtained in both assays corroborated. After the ELISA assay with the pool of sera, the peptides were used in a reactivity test against individual sera from dogs infected with *L*. *infantum*, asymptomatic and symptomatic, in addition, to control canine sera (non-infected, infected with *T*. *cruzi*, *Babesia* sp., and *Ehrlichia* sp.).

### Preparation of crude extract of *L*. *infantum*

The total extract of the parasite was prepared from promastigotes of *L*. *infantum* (MHOM/BR/1974/PP75), which were kept at 24°C in Schneider medium (Sigma-Aldrich, USA), supplemented with 10% inactivated fetal bovine serum, 100 U/mL of penicillin and 100 μg/mL of streptomycin (Gibco/Thermo Fisher Scientific, USA). The parasites kept growing in the logarithmic phase by changing to new culture media every 3 to 4 days. Crude extract was obtained from approximately 5x10^8^ parasites that were washed and resuspended in 1 mL of 1X PBS, followed by lysis through 15 cycles of freezing in liquid nitrogen and thawing at 37°C. The concentration of total parasite proteins was quantified using the Pierce BCA Protein Assay kit (Thermo Fisher Scientific, USA).

### Validation of peptides in Enzyme-linked immunosorbent assay (ELISA)

The recognition pattern of specific IgG antibodies for the peptides and proteins was evaluated by ELISA, using the previously characterized individual samples from the canine serological panel. ELISA assays using as antigens the recombinant proteins; KDDR-plus, Dyn-1, and K39 in addition to crude extract and the peptides derived from the protein rKDDR-plus (K-plus 1 and K-plus 2) and protein rDyn-1 (Dyn-1 peptide) in addition a mixture of these three peptides (Dyn1, K-plus 1 and K-plus 2) were performed as previously described [20]. Briefly, 96-well flat-bottomed microtiter plates (Costar) were coated with 0.1 μg/well of recombinant proteins and of the crude extract and 0.3 μg/well of peptides defined by previous standardizations for 16 hours at 37°C. Sera diluted 1:100 were added to wells in duplicates. Anti-dog IgG conjugated with horseradish peroxidase (Sigma) was used as secondary antibodies diluted 1:2500. Absorbance values of 492 values were read in an automatic ELISA reader (SpectraMax 340 PC, Molecular Devices). The rDyn-1 and rKDDR-plus proteins from *L*. *infantum* were produced and purified as previously described [19,20]. Steven G. Reed (Infectious Disease Research Institute-IDRI, Seattle, Washington) kindly provided the rK39 antigen. The peptide mix was produced by mixing 0.3 μg of each target.

### Statistical analysis

The ELISA results, recorded as optical density (OD) at 492 nm (Molecular Devices, USA), were stored and organized in Microsoft Excel 2010 spreadsheets. Subsequently, the data were distributed using scatter graphics computer software and statistical analysis using GraphPad Prism 8.0 (GraphPad Inc., USA). All serum samples were evaluated in duplicate, with the test result being the mean OD value of these simultaneous determinations. A receiver operating characteristic (ROC) curve was generated for each tested antigen considering the groups of negative dogs (*Babesia* sp., *Ehrlichia* sp., *T*. *cruzi*, and healthy dogs) and positive CanL (symptomatic, asymptomatic, and co-infected) applying the sensitivity values in the ordinate and the complement of specificity in the abscissa. Cut-off values were defined through of the curves for each antigen by choosing the best compromise between sensitivity and specificity associated with the ROC curve [36]. For comparison of the performance between the antigens, the results were expressed by plotting the obtained values in a reactivity index (IR). The IR was established by the ratio between a given sample’s OD and the cutoff value pertaining to microplate/antigen. For the calculation of the cutoff, the best sensitivity and specificity ratio generated in the Prism software was used after plotting all the OD results of each sample. Then, the absorbance of each sample was divided by the cutoff value defined for the plate. All results <1.00 were considered negative and all results >1.00 were considered positive. The ELISA results were compared with the serological status, previously defined by the work’s reference methods (qPCR and parasitological). Each serodiagnostic test was evaluated with respect to sensitivity, specificity, the area under the curve (AUC) considering a 95% confidence interval (95% CI), positive (PPV), and negative (NPV) predictive values, accuracy (AC) and likelihood ratio (LR) [37]. The degree of agreement between the assays was determined by Kappa (κ) index calculated according to Cohen [38] and classified according to Landis and Koch [39] to assess the agreement between ELISA assays and reference methods (qPCR and parasitological): 0.00–0.20 (negligible agreement), 0.21–0.40 (weak), 0.41–0.60 (moderate), 0.61–0.80 (good) and 0.81–1 (excellent).

## Results

### Prediction of immunogenic epitopes of rDyn-1 and rKDDR-plus proteins

**Fig 1:**
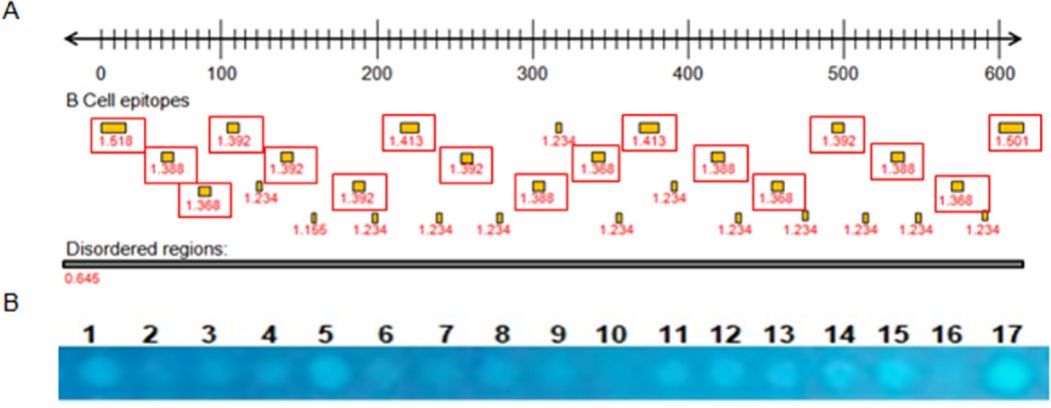
Predictions of B-cell linear e and intrinsically unstructured/disordered regions (entropy) from *L*. *infantum* rDyn-1 gene. **(A)** Each point inside the red boxes represents a B-cell epitopes predicted by BepiPred defined as sequence regions with values above the threshold score of 0.6 and the numbers below indicate the prediction score obtained by each epitope. The black box indicates disordered regions predicted by IUPred with a score of 0.5. Predicted regions as B-cell linear epitopes that are associated with a high degree of structural disorder also exhibit a high antigenicity score, as observed in the graph at the center of the figure. **(B)** Cellulose membrane containing the 17 spots with the peptide sequences derived from the rDun-1 protein selected after bioinformatics analyses visualized by ultraviolet light.

The complete protein sequence of the rDyn-1 protein was subjected to predictions of B cell epitopes and structural disorder. Along the protein sequence, several epitopes with scores above the cut-off and with a high pattern of structural disorder were identified (Fig 1A). However, only sequences that had more than 8 amino acids were considered potential targets and therefore were synthesized in the membrane. Thus, 17 peptide sequences were synthesized in the cellulose membrane (Fig 1B).

**Fig 2:**
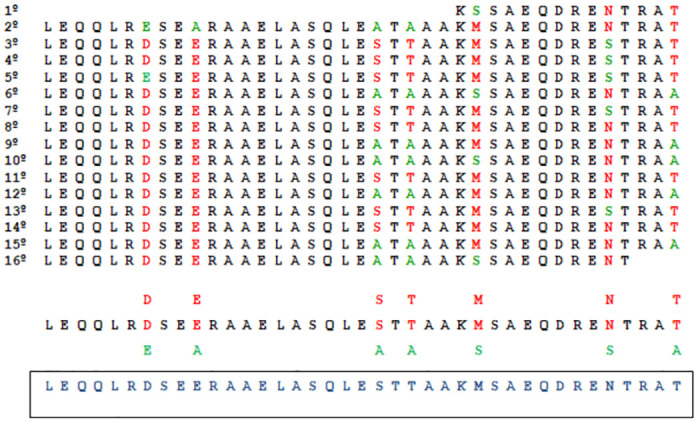
Alignment of the repetitive array of amino acids that make up the rKDDR-plus protein. The 15.3 repetitive blocks containing the 39 amino acids were aligned for better visualization of the least repeated amino acids (green amino acid) and the most repeated (red amino acid) in the seven degenerate sites in black this sequence of the 39 amino acids that make up the 15.3 motifs of the rKDDR-plus protein. In blue is the final sequence used as a template used in the sliding window.

For the identification of potential sequences derived from the complete rKDDR-plus protein, the repetitive block of 39 amino acids that compose the 15.3 sequences of the protein was used. Although the rKDDR-plus protein is characterized by the presence of a single repeating block, these repeats present degenerate regions in seven sites. Therefore, the amino acids that appeared more often in these degenerate sites were defined to compose the sequence of 39 amino acids used as a template for the selection of peptide sequences (Fig 2).

**Fig 3:**
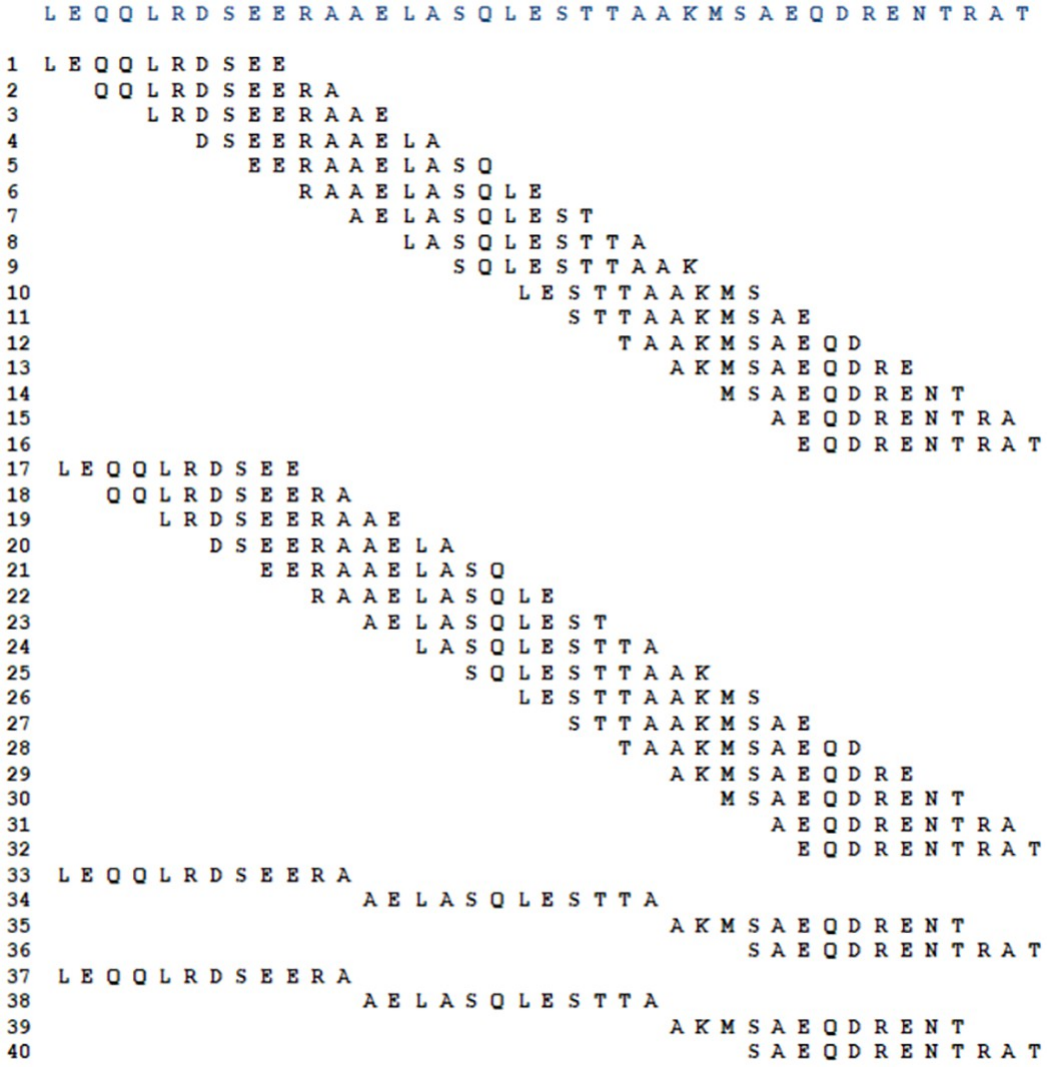
Sliding window technique. The first ten amino acids of the template sequence formed the first set of oligopeptides. Then after a jump of 2 amino acids plus 10 amino acids were selected forming another set of oligopeptides. Following this logic, 16 oligopeptide sequences were selected. To complete the oligopeptide catalog 4 sequences composed of 12 amino acids with random sliding windows were included, completing the 20 sequences of oligopeptides that were synthesized on the membrane in duplicate.

Then, the template sequence was used based on a sliding window of 2 amino acids with a previously defined size that run through the template sequence to be encoded resulting in several smaller sequences of 10 amino acids. After the scan, 16 sequences were selected with the previously defined patterns; another four sequences containing 12 amino acids each were included for synthesis in order to cover a larger screening combination (Fig 3). Finally, 20 peptide sequences derived from the rKDDR-plus protein were elected for synthesis in duplicate, that is, 40 peptides were synthesized on the membrane (S1 Fig).

### Immunoassay with membrane-bound peptides and densitometric analysis

Both the membrane containing the 17 peptides derived from the rDyn-1 protein, as well as the membrane containing the 40 derivatives of the rKDDR-plus protein were subjected to individual incubations on different days with the pool of sera from canines infected with *L*. *infantum* (asymptomatic and symptomatic) and not infected with *L*. *infantum* (healthy control and dogs infected with *T*. *cruzi*, *Babesia* sp. and *Ehrlichia* sp.) to evaluate the discrimination capacity of the selected peptides, through immunoblotting assays. After obtaining the chemiluminescence images of each assay, it was possible to observe points that stand out on the membrane, common in the different incubations, indicating recognition of the antibodies present in the pools used.

**Fig 4:**
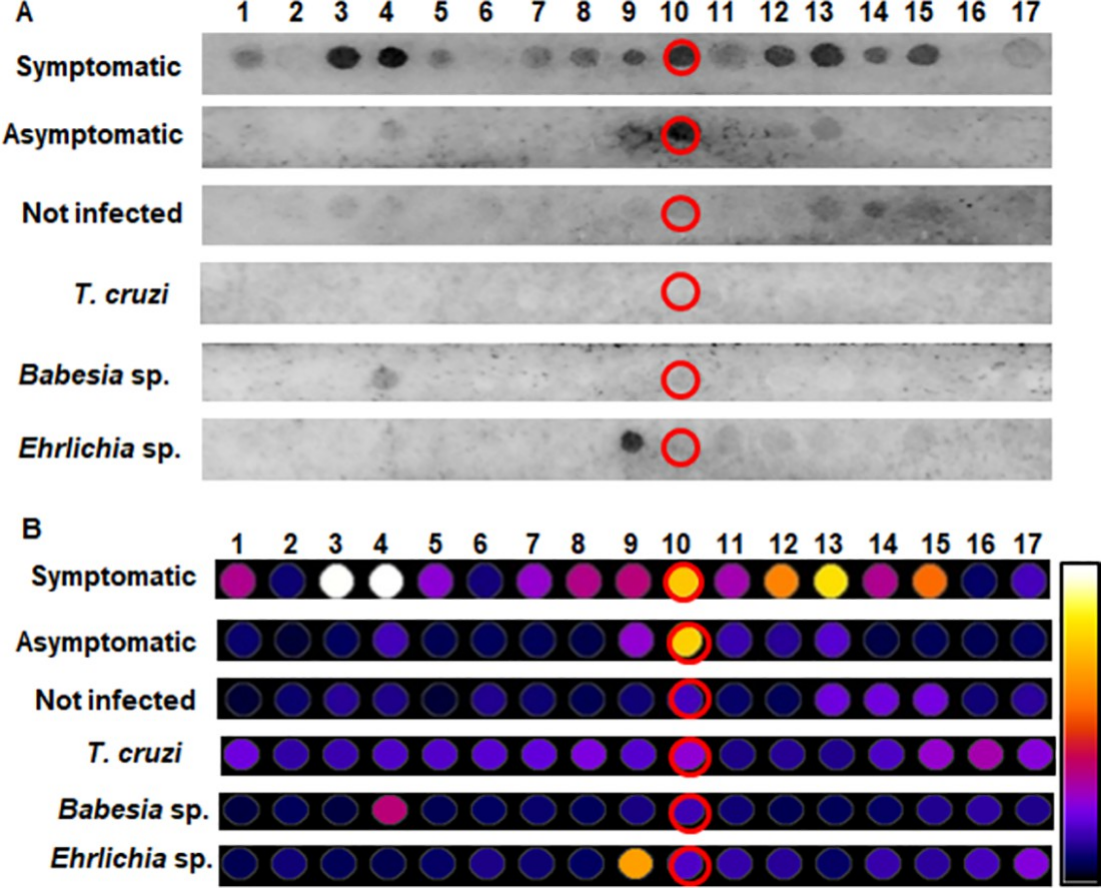
Membrane immunoblotting analysis containing peptides derived from the rDyn-1 protein. **(A)** Chemiluminescence image from spot-synthesis technique to evaluate the reactivity and specificity of rDyn-1-derived peptides incubated with pools of canine sera from symptomatic and asymptomatic infected with *L*. *infantum* and sera from control dogs; healthy dogs without leishmaniasis other pathogens of interest (not infected), dogs infected with *Babesia* sp. and *Ehrlichia* sp. **(B)** Densitometry analysis of previously synthesized peptide spots on the cellulose membrane for selection of the most reactive spot in incubations with pools of dogs infected with *L*. *infantum*, not recognized by sera from dogs without CanL. The closer to the white hue of the scale, the more reactive the spot.

In the membrane that contained the epitopes derived from the rDyn-1 protein, although several peptides were shown to be reactive in the incubation with sera from symptomatic dogs, only 1 spot stood out in the incubation with asymptomatic sera. This same spot showed no reactivity against incubations with the sera of the control groups (healthy dogs, with *T*. *cruzi*, *Babesia* sp., and *Ehrlichia* sp.) proving to be a promising target to be soluble synthesized (Fig 4A). Densitometry analyzes were performed to validate the selection of the peptide at position 10 (Dyn-1 peptide), as it presents a high recognition signal with the pool of sera from dogs infected with *L*. *infantum* (asymptomatic and symptomatic) and low recognition with sera from control dogs (dogs not infected with *L*. *infantum*, infected with *T*. *cruzi*, *Babesia* sp. and *Ehrlichia* sp.) (Fig 4B).

**Fig 5:**
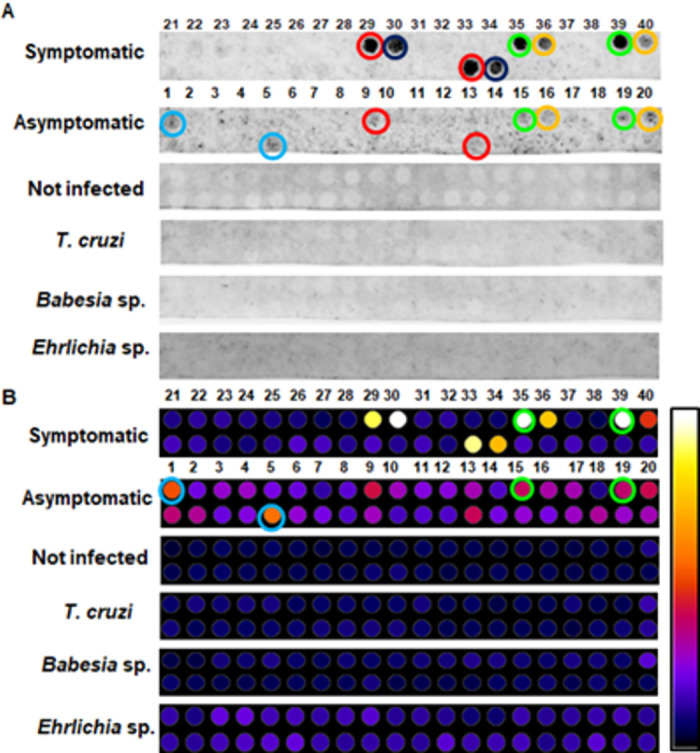
Analysis of the membrane containing peptides derived from the rKDDR-plus protein. **(A)** Chemiluminescence image from membrane immunoblotting assays incubated with pools of sera from symptomatic and asymptomatic dogs infected with *L*. *infantum* and sera from control dogs; healthy dogs without leishmaniasis other pathogens of interest (not infected), dogs infected with *Babesia* sp. and *Ehrlichia* sp. Red, green, and yellow spots were lit for both groups of infected dogs. Dark blue spot lit only for the pool of symptomatic dogs and light blue spot lit only for the pool of asymptomatic sera. (B) Densitometric analysis for selection of the most reactive spots in incubations with pools of dogs infected with *L*. *infantum*, not recognized by sera from dogs without CanL. The closer to the white hue of the scale, the more reactive the spot. Green circle; reactive spots with symptomatic and asymptomatic sera. Blue circle; more reactive spots only with asymptomatic sera.

Twenty peptide sequences derived from the rKDDR-plus protein were screened by the scan. These sequences were synthesized in duplicate to reinforce the immunoblotting results. Therefore, it is possible to observe 40 peptides on the membrane, facilitating the identification and localization of duplicated peptides (S1 Fig). From the images obtained from the chemiluminescence of the membrane immunoblotting (Fig 5A), it is possible to observe that none of the spots showed reactivity during incubation with the control sera pools (healthy dogs, *T*. *cruzi*, *Babesia* sp., or *Ehrlichia* sp.). This result indicates that the peptides do not present cross-reactivity with other evaluated parasites or even with sera from healthy dogs. On the other hand, when incubating the membrane with pools of sera with *L*. *infantum*, some spots and its duplicates were reactive for both clinical forms. Spots 13 and 29 (duplicate) red circle, spots 35 and 39 (duplicate) green circle, and spots 36 and 40 (duplicate) yellow circle were brightly during incubation with the symptomatic pool of sera and were slightly reactive during incubation with the pool of asymptomatic sera. Spot 14 (dark blue) and its duplicate (spot 30) were strongly recognized by the symptomatic pool and did not light up when incubated with an asymptomatic pool. When analyzing the membrane incubated with the asymptomatic pool the spot 5 and 21 (duplicate); light blue circle, were recognized, but did not show any reactivity with the pool of symptomatic sera. However, after normalizing the duplicates of the spots and analyzing the scores obtained in the densitometry analyses (Fig 5B) were selected the spots 35 and 39 (duplicate) green circle. Because it presented the highest recognition signal both with sera from symptomatic dogs and sera from dogs asymptomatic dogs. In addition, spot 5 and 21 (duplicate) light blue circle also were selected because it showed better recognition signal with antibodies from asymptomatic dogs. The other spots were not selected for synthesis in this work, because although they were recognized by the antibodies present in the sera pools of dogs with *L*. *infantum*, their scores were lower.

### Soluble synthesis of peptides and mass spectrometry (MALDI/TOF)

After immunoblotting and densitometry analysis, three peptide sequences were synthesized in the solid phase (SPFS). The peptides were produced anchored to polymeric support that allowed their production in soluble format [35] (Table 1). To confirm whether the synthesized soluble peptides were compatible with the expected sequences, the crude products were subjected to mass spectrometry analysis by MALDI in TOF/MS/MS (S2 Fig) mode, which confirmed the expected molecular mass and, therefore, the compatibility with the expected peptide sequences.

**Table 1:** Selected peptic sequences.

Peptide	Predicted molecular mass (g/mol)	Molecular mass MALDI TOF/MS/MS (g/mol)	Sequence	Source protein
Dyn-1	120129	1201.6876	I-K-R-D-D-R-K-D-D	rDyn-1
K-plus 1	1377.63	1378.6980	A-K-M-S-A-E-Q-D-R-E-N-T	rKDDR-plus
K-plus 2	1101.54	1102.6672	E-E-R-A-A-E-L-A-S-Q	rKDDR-plus

### Comparative efficacy between rDyn-1 and KDDR-plus precursor proteins and their more immunogenic peptide derivatives

**Fig 6:**
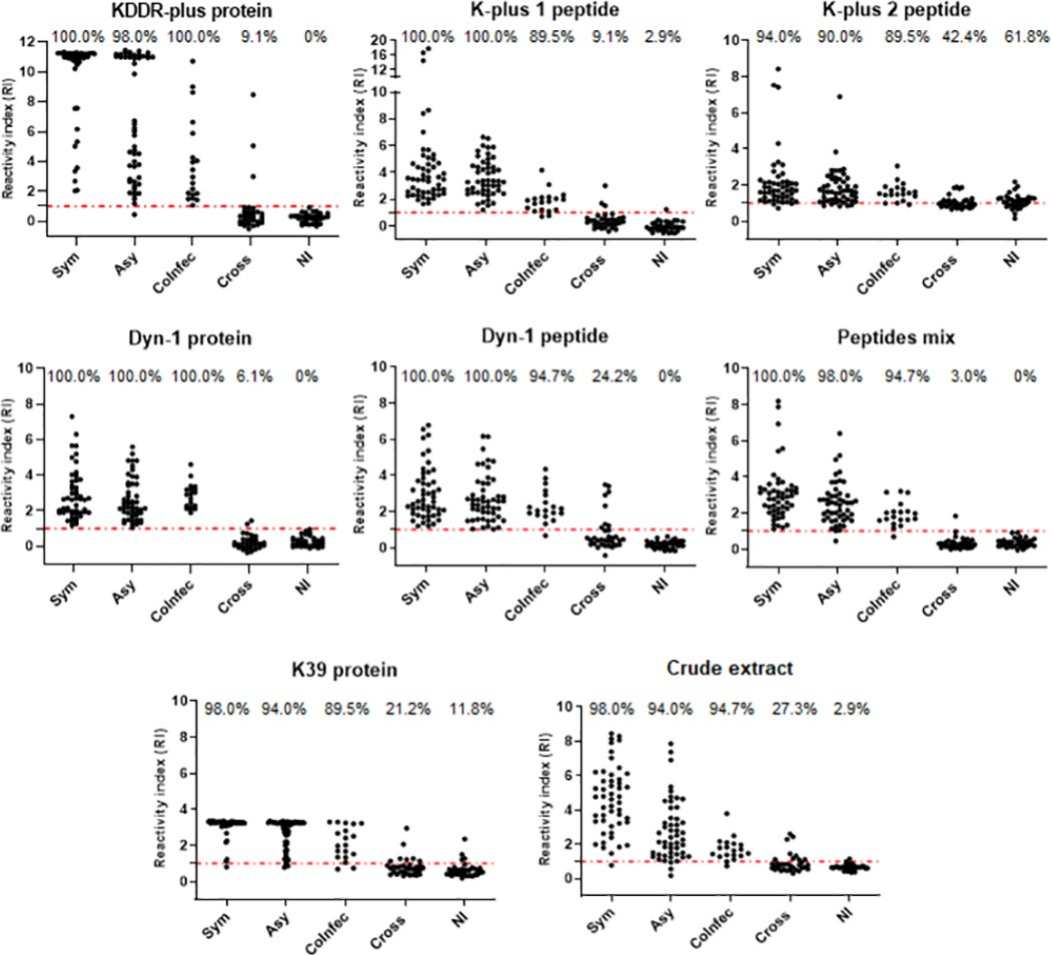
Comparing the reactivity of canine sera using rKDDR-plus, rDyn-1 and K39 proteins, their derived peptides K-plus 1, K-plus 2, Dyn-1 and mixtures of these three peptides and the crude extract of *L*. *infantum* in an ELISA protocol. Reactivity index results obtained under *Leishmania* complete recombinant proteins Dyn-1 and KDDR-plus, the synthetic peptides derived from each recombinant protein (Dyn-1, K-plus 1 and K-plus 2 peptides), a mixture (Mix peptides) of 0.3 μg/well of the three peptides and control antigens rK39 and crude extract against a canine serological panel composed by serum from infected dogs presenting symptoms (Sym), infected asymptomatic dogs (Asy), dogs co-infected with *Leishmania* and *Babesia* sp. (CoInfec), dogs with other infections (*Babesia* sp., *Ehrlichia* sp. and T. cruzi) (Cross) and not infected (NI). The index above each column in the plot indicates the percentage of samples that are above the cut-off.

ELISA experiments were performed using the complete recombinant proteins Dyn-1 and KDDR-plus, the synthetic peptides derived from each recombinant protein (Dyn-1, K-plus 1, and K-plus 2 peptides), and control antigens rK39 and crude extract against a canine serological panel to identify which of them offered highest performances. Furthermore, a pool containing a mixture (Mix peptides) of 0.3 μg/well of each peptides evaluated in this work was also included. Fig 6 illustrates the performance parameters and reactivity index (RI) distributions obtained by ELISA tests using the recombinant proteins, peptides and control antigens performed on 119 samples from dogs with CanL being 50 symptomatic dogs, 50 asymptomatic and 19 coinfected (*Leishmania* and *Babesia* sp.); 33 dogs with other infections (*Babesia* sp., *Ehrlichia* sp. and *T*. *cruzi*) and 34 samples from not infected dogs.

The diagnostic performance of each antigen evaluated is summarized in Table 2. rDyn-1 protein was the only antigen able to determine all cases of dogs carrying *L*. *infantum* (asymptomatic, symptomatic and co-infected) yielding an overall sensitivity of 100%. The mixture of peptides, the Dyn-1 and K-plus 1 peptides and the rKDDR-plus protein were able to identify 100% of the dogs considered symptomatic, but failed to identify asymptomatic or co-infected dogs. Thus, the general sensitivity of the rKDDR-plus protein and the Dyn-1 fi peptide was 99.16% and the peptide mixture and the K-plus 1 peptide was 98.32%. The other antigens were not able to determine with 100% none of the groups of infected dogs. Despite not having recognized all dogs positive for CanL, the peptide mixture showed the best specificity result = 98.51% in addition to the highest LR value (65.87) among all the antigens evaluated in this work. The proteins rDyn -1 and rKDDR-plus followed by peptide K-plus 1 also showed satisfactory results in relation to specificity 97.01%, 95.52% and 94.03%, respectively, and LR 33.50, 22.15 and 16.47, respectively. Based on these results, it is possible to suggest the use of a mixture of peptides in addition to K-plus 1 and Dyn-1 peptides as new markers for the diagnosis of CanL.

**Table 2:** Serological evaluation of antigens for diagnosis of leishmaniasis.

Antigen	Sensitivity (CI 95%; N = 119)	Sensitivity (%)			Specificity (CI 95%; N = 67)	Specificity (%)					LR
		Sym. (N = 50)	Asy. (N = 50)	CoInfec (N = 19)		Bab. (N = 7)	*Ehr*. (N = 6)	*T*. *cruzi* (N = 20)	NI (N = 34)	AC (%)	
**rDyn-1 protein**	100.0 0.969 to 1.00	100.0	100.0	100.0	97.01 0.8963 to 0.996	71.43	100.0	100.0	100.0	98.92	33.50
**Mix peptides**	98.32 0.940 to 0.998	100.0	98.00	94.74	98.51 0.9196 to 0.999	100.0	83.33	100.0	100.0	98.39	65.87
**rKDDR-plus protein**	99.16 0.954 to 0.999	100.0	98.00	100.00	95.52 0.8747 to 0.990	71.43	100.0	95.00	100.00	97.85	22.15
**K-plus 1 peptide**	98.32 0.940 to 0.998	100.0	100.0	89.47	94.03 0.8541 to 0.983	100.0	66.67	95.00	97.06	96.77	16.47
**Dyn-1 peptide**	99.16 0.954 to 0.999	100.0	100.0	94.74	88.06 0.7782 to 0.947	100.0	100.0	60.00	100.0	95.16	8.3
**Crude extract**	95.8 0.904 to 0.986	98.00	94.00	94.74	85,07 0.7426 to 0.926	71.43	100.0	65.00	97.06	91.94	6.4
**rK39**	94.96 0.893 to 0.981	98.00	94.00	89.47	83.58 0.7252 to 0.915	71.43	66.67	85.00	88.24	90.86	5.78
**K-plus 2 peptide**	91.6 0.8509 to 0.9590	94.00	90.00	89.47	47.76 0.3540 to 0.603	57.14	33.33	65.00	38.24	75.81	1.75

Abbreviations: CI: confidence interval, Sym.: symptomatic, Asy.: asymptomatic, CoInfec: coinfected, Bab.: *Babesia* sp., Ehr: *Ehrlichia* sp., NI: not infected, AC: accuracy LR: likelihood.

**Fig 7:**
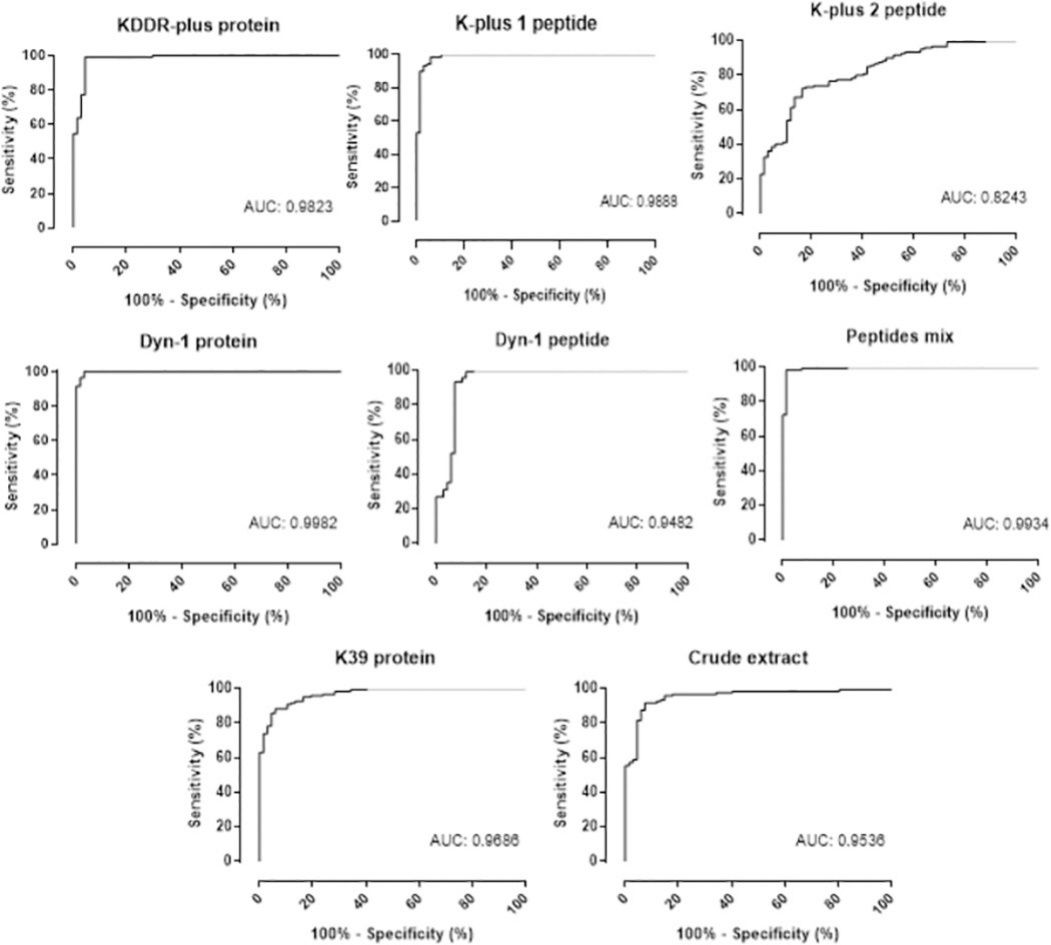
ROC curve analysis of the area under the curve (AUC), considering the results from ELISA. Comparation of the ROC curve obtained employing *Leishmania* complete recombinant proteins Dyn-1 and KDDR-plus, the synthetic peptides derived from each recombinant protein (Dyn-1, K-plus 1 and K-plus 2 peptides), a mixture (Mix peptides) of 0.3 μg/well of the three peptides and control antigens rK39 and crude extract was established by GraphPad Prism 8.0 using serum samples of negative and positive samples in each plate. Abbreviations: (AUC) area under curve.

ROC curves were generated for each antigen tested to determine the test sensitivity and specificity (Fig 7). Analysis of the area under the curve (AUC) confirmed the superior performance of the rDyn-1 protein and the peptides mixture (AUC = 0.9982 and 0.9934, respectively) in comparison to the other proteins and peptides evaluated; K-plus 1 peptide (AUC = 0.9888), rKDDR-plus protein (AUC = 0.9823), rK39 (AUC = 0.9686), crude extract (AUC = 0.9536), Dyn-1 peptide (AUC = 0.9482) and K-plus 2 peptide (AUC = 0.8243).

The results obtained in the ELISA tests confirmed that the peptide mixture in addition to the rDyn-1 protein offers the best diagnostic performances for CanL. When we analyze the Kappa agreement index of 0.965 and 0.997, respectively, generating an excellent level of agreement. However, the rKDDR-plus protein and its derived peptide K-plus 1 do not leave anything to be desired, with a Kappa index > 0.9 and a degree of agreement considered excellent. Although the Dyn-1 peptide and the crude extract also showed a degree of agreement considered excellent, their Kappa values were less than 0.9. The rK39 protein and the K-plus 2 peptide, on the other hand, presented the lowest Kappa values and, consequently, the lowest agreement levels among the analyzed antigens. Of note, both the precursor proteins rDyn-1 and rKDDR-plus and the peptides derived from them; K-plus 1 and Dyn-1 together with the mixture of peptides proved to be good targets to be used in the diagnosis of CanL. The rDyn-1 protein showed the best performance in CanL diagnosis (98.92% accuracy), followed the peptide mixture (98.39% accuracy), the rKDDR-plus protein (97.85%), by the K-plus 1 and Dyn-1 peptides (accuracy of 96.77% and 95.16%, respectively), followed by the control antigens crude extract and rK39 (accuracy of 91.94% and 90.86%, respectively) and finally by the K-plus 2 peptide (accuracy of 75.81%) (S1 Table).

## Discussion

In recent years, there has been a progressive increase in studies aimed at finding a more accurate diagnosis for CanL. A good part of these studies, if not the majority, is focused on the identification of highly antigenic molecules to the antibodies produced by the host during infection by the parasite responsible for the disease. The identification of infected dogs, regardless of clinical status, is necessary as part of the disease prevention and control measures in the canine population and consequently in the human population. However, dogs that have been infected by the parasite confirmed by valid diagnostic methods, but do not show any clinical signs compatible with the disease, are considered a potential reservoir host, mainly in endemic areas for zoonotic leishmaniasis, which may lead to human infection [40]. Therefore, *Leishmania*-infected dogs especially the asymptomatic dogs represent a challenge for the control of leishmaniasis in the human population, because these dogs have a potential role in maintaining *L*. *infantum* infection and probably in establishing the parasite’s domestic transmission cycle in disease endemic areas [41].

In this context, highly sensitive and specific antigens, capable of being used in simple, quick tests and with easy adaptability in the field, dispensing with the use of expensive equipment and specialized technicians, are at the heart of these discussions. The technology for the production of recombinant molecules has helped a lot in the search for new antigens for both leishmaniasis and other diseases [42]. Our previous investigations demonstrated that recombinant antigens from *L*. *infantum* have high sensitivity and specificity in the diagnosis of CanL, especially in dogs without clinical signs of the disease, asymptomatic ones [19,20]. In this work, the amino acid sequences of the two proteins previously described by our research group were screened and three oligopeptides were evaluated for diagnosis of *L*. *infantum*-infected dogs. Furthermore, the three peptides were combined into a mixture, which was also evaluated in ELISA assays. The rKDDR-plus and rDyn-1 proteins carry immunodominant epitopes useful for the serological diagnosis of CanL [19,20]. In this study, the complete amino acid sequences of these two proteins were unraveled in order to locate unique epitopes responsible for the positive performance of both for diagnosing CanL. The screening by immunoblotting assays identified one peptide derived from rDyn-1 protein and two derived from rKDDR-plus protein with good recognition with pools of dogs infected with *L*. *infantum*, symptomatic and asymptomatic, not being recognized by sera from dogs without *L*. *infantum* (dogs healthy or infected with other pathogens). The results suggest that sequences derived from rDyn-1 and rKDDR-plus would be useful for developing new antigens that could improve the sensitivity and specificity of diagnostic tests. This result may be a consequence of initial screening in silico of the prediction of immunodominant B-cell epitopes and structural disorder characteristics for selecting only sequences above the determined cut-off. On the other hand, it is worth mentioning the immunogenic property of the repeats of the 39 amino acid sequence of block 15.3 present in the KDDR-plus protein by itself [20].

The rKDDR-plus proved to be advantageous in terms of specificity, proving to be excellent to discriminate dogs without infection by *Leishmania*, that is healthy dogs or dogs that have a pathology other than leishmaniasis that could cause a false positive result [20]. Corroborating the findings of Siqueira et al. (2021), in this work, our results show a low cross-reactivity index of rKDDR-plus, maintaining the ability to identify dogs with CanL. One of the peptides derived from rKDDR-plus, the K-plus 1 peptide, presented a diagnostic performance similar to its precursor with good sensitivity of 98.32% (K-plus 1) and 99.16% (rKDDR-plus) and specificity of 94.03% (K-plus 1) and 95.52% (rKDDR-plus).

On the other hand, the second peptide derived from rKDDR-plus (K-plus 2) obtained a lower than expected diagnostic quality in ELISA assays with individual sera. The immunoblotting assay showed that the K-plus 2 peptide was able to react with asymptomatic sera, but was not reactive with sera from symptomatic dogs. When the K-plus 2 peptide was analyzed in ELISA assays, it was just as capable of identifying asymptomatic as well as symptomatic dogs. However, the biggest question related to the low performance of K-2 plus is its cross-reactivity index, which, unlike its precursor, presented a low specificity index in the ELISA assays. In the immunoblotting assays, K-plus 2 did not recognize any sera from dogs with other pathologies. However, in the ELISA assays, this result was not maintained, making the K-plus 2 performance poor. Its diagnostic accuracy was only 75.81%, below all the antigens evaluated in this work, including the crude extract control antigens (91.94% accuracy) and rK39 (90.86% accuracy), presenting results that indicate that it is not a good diagnostic marker for CanL when used individually. It is known that there is a possibility that different approaches to the same molecule can influence its performance, as in the case of K-plus 2, modifying its ability to bind to antibodies derived from dogs [43]. The conformation and disposition of the oligopeptides in the immunoblotting membrane and in ELISA assays are different and this means that the same molecule can expose or hide some relevant epitope [43]. Another justification for this divergence is the increase in the number of sera in the ELISA assays in relation to the number of sera applied in the assembly of the serological pool used in immunoblotting. This issue was considered, by performing a primary ELISA assay using the same pool of sera used in the immunoblotting assays. Thus, it was seen that there were no negative divergences between the findings of each test, except for the fact that the K-plus 2 peptide was able to recognize both symptomatic and asymptomatic dogs, which is why it was maintained in this study.

The amino acid sequence from the rDyn-1 protein was also studied. Previous studies have reported a satisfactory performance regarding rDyn-1, a protein belonging to the protein superfamily dynamins related to several processes of membrane dynamics and functioning, for the diagnosis of CanL [19]. The rDyn-1 protein stood out previously for its excellent ability to identify dogs without clinical signs compatible with the disease, and that present positive diagnostic in a combination of tests (serological, molecular, and/or parasitological) characterized as asymptomatic (sensitivity = 100%) [19]. Among all the antigens (proteins, peptides, and crude extract) evaluated in this work, the rDyn-1 protein showed the best diagnostic accuracy (98.92%) maintaining the high ability to identify dogs with CanL (symptomatic, asymptomatic, and co-infected) (sensitivity = 100%) corroborating with the previous studies in relation to the identification of asymptomatic dogs. It is noteworthy that in the previous work, rDyn-1 was not able to correctly identify all cases of dogs with clinical signs of the disease, the symptomatic dogs (sensitivity = 92%) contrary to what was observed in the current work. This fact can be explained by the increase in the protein mass used in this work. In the previous work, the ELISA plates were sensitized with 50ng/well of the protein and in this work, 100ng/well was used. Here the rDyn-1 also presented a smaller cross-reaction index with dogs without CanL or with other diseases (specificity = 97.01%). However, only one peptide derived from rDyn-1 protein (Dyn-1 peptide) was selected to be tested in the ELISA assays. This peptide was the only one that was shown to be reactive in the incubation with the pool of sera from dogs infected with *L*. *infantum* (asymptomatic and symptomatic) presenting low or no degree of recognition with sera from control dogs (dogs not infected with *L*. *infantum*, infected with *T*. *cruzi*, *Babesia* sp. and *Ehrlichia* sp.) in the immunoblotting assay. As well as in the immunoblotting assays, the Dyn-1 peptide was also able to identify all symptomatic and asymptomatic dogs in ELISA assays, but it was not able to determine all co-infections (*Leishmania* and *Babesia* sp.) presenting a sensibility de 99.16% and a moderate index of cross-reaction mainly with sera from dogs infected with *T*. *cruzi* (specificity = 88.06%).

Additionally, a fourth peptide, composed of a homogeneous mixture of the three peptides (Dyn1, K-plus 1, and K-plus 2) evaluated here, was included for preliminary analysis in ELISA assays. The peptide mix, surprisingly, showed excellent diagnostic performance, as well as the rDyn-1 protein. Although the rDyn-1 protein showed the best accuracy (98.92%) and sensitivity (100%) among all the antigens analyzed in this study, the peptides mix showed the highest specificity values (98.51%) and likelihood ratio (65.87) than the rDyn-1 protein (specificity = 97.01 and likelihood ratio 33.50). This result shows the potential of this mixture as a serological marker for the diagnosis of CanL. We saw that the performance of the K-plus 2 peptide individually was lower than expected. Although, when present in the peptide mixture, the performance of this mixture is attractive. This shows that sometimes individual peptides can present varied performance results. However, when used together and in previously studied proportions, they can reach an accuracy equal to or greater than the sum of the individualized peptides. This reinforces the theory that antigenic mixtures with multi-epitopes or chimeric may be the way to achieve impeccable diagnostic performance [44–46].

Peptide synthesis is another introduced alternative for researchers to search for antigenic targets for the disease [47]. Synthetic peptides are excellent antigens to be used in several serological tests, including ELISA [48]. These small portions are designed from amino acid sequences of potentially antigenic proteins already characterized for the diagnosis of the disease. Synthetic peptides have an advantage over proteins because the fragments are selected based on a specific criterion or characteristic and allow the assembly of a new molecule composed of several epitopes of interest [49]. Therefore, these sequences are composed of epitopes that are more singular and directed to the target of interest. With this, it is possible to build a molecule, composed of different sequences with specific characteristics that, when used together, provide higher diagnostic accuracy than that obtained with their individual use. This approach has been gaining strength in the diagnosis of CanL with the use of chimeric antigens or antigenic mixtures composed of multiple epitopes to be used as antigens in serological tests [50].

It is noteworthy that in this work the rK39 and crude extract antigens were included as a control because they are widely used in the serological diagnosis of CanL in Brazil and worldwide. However, using the total extract of the parasite has fallen into disuse in view of its high rates of cross-reactivity reactions with other pathogens that often coexist in endemic areas, leading to inconsistent results [51,52]. In turn, the limitation of the rK39 antigen is mainly related to the highly varied performance between different regions of the world, making its use limited to geographic regions where previous studies have shown good performance [53,54]. As reported in the literature, the crude extract and rK39 antigen showed limited results for specificity (85.07% and 83.58%, respectively) resulting in an accuracy of 91.94% and 90.86%, respectively increasingly discouraging the use of these targets as markers for the diagnosis of CanL.

In the search for targets that help in the diagnosis of diseases such as leishmaniasis, many works have focused on a deeper study of genes and their transcripts and protein products involved in important cellular processes, as in the case of the two recombinant proteins and their peptides used in this work. The search for new antigens is a constant search, which must be done to reach a diagnosis that presents 100% sensitivity and specificity. With this, it is expected that the development of more accurate diagnostic tests, which present sufficient sensitivity to correctly identify all cases of leishmaniasis. Antigens with satisfactory specificity are also expected allowing the correct discrimination of case negatives and avoiding false positive results. It is convenient to remember that the early diagnosis of CanL can help in the control of the infection in dogs and, consequently, in the reduction of human infection.

In this work, oligopeptides derived from proteins previously described in the literature, with immunogenic properties were selected to build a multi-peptide molecule for application in diagnostic tests for CanL. This data should be considered as providing proof-of-concept or a preliminary study. Both the rDyn-1 and rKDDR-plus proteins and the peptides derived from them Dyn-1 and K-plus 1, respectively, as well as the mixture of peptides showed high performance and can be applied as markers for the diagnosis of CanL. The mixture of peptides deserves to be evaluated with different compositions and concentrations. Testing with other peptide ratios and compositions is recommended for a fairer comparison of the results obtained so far. The influence of the K-2 plus peptide in this mixture must also be evaluated. It should also be considered as a limiting factor of this work the absence of higher and more diverse serological panels to include more samples most representative of the canine population both for CanL and for cross-reactive diseases.

In conclusion, we identified peptides to compose a molecule with multiple epitopes to improve CanL serodiagnosis. Our findings indicate that the peptides studied here may be useful to compose this molecule and allow the detection of symptomatic dogs as well as asymptomatic dogs. Based on the results obtained so far in this study, we hope to contribute to the control of leishmaniasis by developing tests that are more efficient and encouraging the production of new technologies not only for leishmaniasis but also for other infectious diseases. Antigens with multiple epitopes can be used in enzyme-linked immunosorbent assays (ELISA), lateral flow assays, biosensors, and cellular assays. Therefore, in a future perspective, it is expected that the peptides selected and validated here by serological ELISA assays be used on other diagnostic platforms, which have a greater applicability in diagnostic screening in the field.

## Supporting information

S1 Fig Cellulose membrane with peptide sequences derived from the rKDDR-plus protein visualized by ultraviolet light. Sixteen peptides selected after the scan were synthesized in sequence on the membrane (numbers 1 to 16 red), and then duplicates of these sequences were also synthesized on the membrane (numbers 17 to 32 green). Then, the four sequences containing twelve amino acids each were included in the membrane (numbers 33 to 36 blue), followed by their duplicates (numbers 37 to 40 purple).
(TIF)

S2 Fig MALDI-TOF MS peptide elution analysis. **(A)** MALDI-TOF MS analysis of the peak from Dyn-1 peptide. **(B)** MALDI-TOF MS analysis of the peak from K-plus 1. (C) MALDI-TOF MS analysis of the peak from K-plus 2.
(TIF)

S1 Table Comparison of performance of diagnostic tests for CanL using a validation serum panel. (PDF)

S1 File ELISA data obtained in this study. Data used to make ELISA figures and tables.
(PDF)

S2 File STARD checklist. Reports of studies of diagnostic accuracy.
(PDF)
