# Supplementary figures and images for: Immunogenic mapping of rDyn-1 and rKDDR-plus proteins and selection of oligopeptides by immunoblotting for the diagnosis of Leishmania infantum-infected dogs
Source: PLoS Negl Trop Dis. 2023 Aug 4;17(8):e0011535. doi: 10.1371/journal.pntd.0011535 (PMC10442149; doi:10.1371/journal.pntd.0011535)

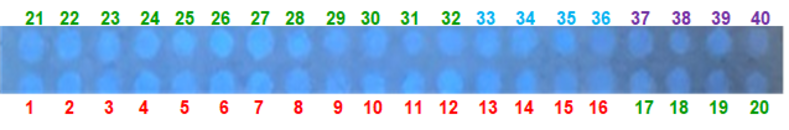

Supplement: S1 Fig — Sixteen peptides selected after the scan were synthesized in sequence on the membrane (numbers 1 to 16 red), and then duplicates of these sequences were also synthesized on the membrane (numbers 17 to 32 green). Then, the four sequences containing twelve amino acids each were included in the membrane (numbers 33 to 36 blue), followed by their duplicates (numbers 37 to 40 purple). (TIF) [file pntd.0011535.s001.tif]

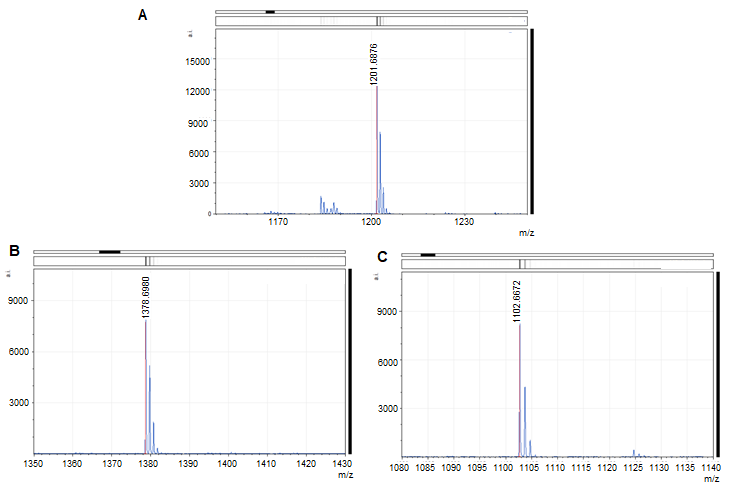

Supplement: S2 Fig — (A) MALDI-TOF MS analysis of the peak from Dyn-1 peptide. (B) MALDI-TOF MS analysis of the peak from K-plus 1. (C) MALDI-TOF MS analysis of the peak from K-plus 2. (TIF) [file pntd.0011535.s002.tif]
