# Supplementary material for: Immunogenic mapping of rDyn-1 and rKDDR-plus proteins and selection of oligopeptides by immunoblotting for the diagnosis of Leishmania infantum-infected dogs
Source: PLoS Negl Trop Dis. 2023 Aug 4;17(8):e0011535. doi: 10.1371/journal.pntd.0011535 (PMC10442149; doi:10.1371/journal.pntd.0011535)
Supplement: S1 Table — (PDF) [file pntd.0011535.s003.pdf]

Table 1. Comparison of performance of diagnostic tests for CanL using a validation serum panel.

Antigen	Positive samples			Negative samples				PPV (%)	NPV (%)	K	Level of Agreement
	Sym.	Asy.	CoInfec	Bab.	Ehr.	*T. cruzi*	NI				
**rDyn-1 protein**	50/50	50/50	19/19	5/7	6/6	20/20	34/34	98.35	100.0	0.977	Excellent
**Mix peptides**	50/50	49/50	18/19	7/7	5/6	20/20	34/34	99.15	97.06	0.965	Excellent
**rKDDR-plus protein**	50/50	49/50	19/19	5/7	6/6	19/20	34/34	97.52	98.46	0.953	Excellent
**K-plus 1 peptide**	50/50	50/50	17/19	7/7	4/6	19/20	33/34	96.69	96.92	0.930	Excellent
**Dyn-1 peptide**	50/50	50/50	18/19	7/7	6/6	12/20	34/34	93.65	98.33	0.893	Excellent
**CSA**	49/50	47/50	18/19	5/7	6/6	13/20	33/34	91.94	91.94	0.822	Excellent
**rK39**	49/50	47/50	17/19	5/7	4/6	17/20	30/34	91.13	90.32	0.798	Good
**K-plus 2 peptide**	47/50	45/50	17/19	4/7	2/6	13/20	13/34	75.69	76.19	0.429	Moderate

Abbreviations: CSA: crude soluble antigen, Sym.: symptomatic, Asy.: asymptomatic, CoInfec.: coinfected, Bab.: *Babesia* sp., Ehr.: *Ehrlichia* sp., NI: not infected, PPV: positive predictive value, NPV: negative predictive value, K: index kappa.
