# Supplementary material for: Immunogenic mapping of rDyn-1 and rKDDR-plus proteins and selection of oligopeptides by immunoblotting for the diagnosis of Leishmania infantum-infected dogs
Source: PLoS Negl Trop Dis. 2023 Aug 4;17(8):e0011535. doi: 10.1371/journal.pntd.0011535 (PMC10442149; doi:10.1371/journal.pntd.0011535)
Supplement: S1 File — Data used to make ELISA figures and tables. (PDF) [file pntd.0011535.s004.pdf]

	Dyn-1 protein		Mix peptides		KDDR-plus protein		K-plus 1 peptide		Dyn-1 peptide		CSA		K39		K-plus 2 peptide	
**Cutoff**	0,4786		0,3955		0,3262		0,1822		0,4907		0,4064		1,003		0,3947	
**Area**	0.9982		0.9934		0.9823		0.9888		0.9482		0.9536		0.9686		0.8243	
**99% CI**	0.9953 to 1.000		0.9840 to 1.000		0.9635 to 1.000		0.9742 to 1.000		0.9060 to 0.9904		0.9224 to 0.9847		0.9481 to 0.9892		0.7640 to 0.8846	
**Sens.**	100		98.32		99.16		98.32		99.16		95.8		94.96		91.6	
**99% CI**	0.9695 to 1.000		0.9406 to 0.9980		0.95451 to 0.9998		0.9406 to .,9980		0.9541 to 0.9998		0.9047 to 0.9862		0.8935 to 0.9813		0.8509 to 0.9590	
**Espc.**	97.01		98.51		95.52		94.03		88.06		85.07		83.58		47.76	
**99% CI**	0.8963 o 0.9964		0.9196 to 0.9996		0.8747 to 0.9907		0.8541 to 0.9835		0.7782 to 0.9470		0.7426 to 0.9260		0.7252 to 0.9151		0.3540 to 0.6033	
	**OD**	**RI**	**OD**	**RI**	**OD**	**RI**	**OD**	**RI**	**OD**	**RI**	**OD**	**RI**	**OD**	**RI**	**OD**	**RI**
Sint-100	0,91455	1,9108859	1,21885	3,0817952	3,58665	10,995248	0,84075	4,6144347	1,7839	3,6354188	1,88165	4,6300443	3,2715	3,2617	0,8545	2,1649354
Sint-102	0,6733	1,4068115	0,4775	1,2073325	1,0976	3,3648069	0,42045	2,307629	0,6758	1,3772162	0,84995	2,0914124	2,1832	2,17667	0,4277	1,0836078
Sint-103	0,914	1,9097367	0,44195	1,1174463	0,6624	2,030656	0,36755	2,0172887	1,0852	2,2115345	0,7914	1,9473425	1,12585	1,1224826	0,4119	1,0435774
Sint-104	1,29215	2,6998537	0,8039	2,0326169	3,6062	11,055181	0,6066	3,3293085	1,0501	2,1400041	1,5045	3,7020177	3,25505	3,2453141	0,6234	1,5794274
Sint-105	0,699	1,4605098	0,5121	1,2948167	0,679	2,0815451	0,357	1,9593853	0,8572	1,7468922	0,6017	1,480561	1,23585	1,2321535	0,512	1,2971877
Sint-106	1,2759	2,6659005	2,14615	5,4264223	3,69925	11,340435	1,57705	8,6555982	1,7471	3,5604239	2,1715	5,3432579	3,1711	3,1616152	0,8067	2,0438308
Sint-107	1,28465	2,684183	0,9322	2,3570164	3,68375	11,292918	0,42205	2,3164105	0,9338	1,9029957	1,6327	4,0174705	3,2159	3,2062812	0,4412	1,117811
Sint-108	0,80765	1,6875261	1,0274	2,5977244	0,8772	2,6891478	0,93005	5,1045554	1,5752	3,210108	0,753	1,8528543	0,811	0,8085743	0,8383	2,1238916
Sint-109	0,9398	1,963644	0,5276	1,3340076	2,01075	6,1641631	0,49905	2,7390231	0,9994	2,0366823	0,81415	2,0033219	3,0556	3,0464606	0,43245	1,0956423
Sint-110	0,9062	1,8934392	1,3027	3,2938053	3,59965	11,035101	0,4826	2,6487377	1,30005	2,6493784	3,01125	7,4095719	3,32755	3,3175972	0,5624	1,4248797
Sint-111	0,6849	1,4310489	1,11415	2,817067	3,6481	11,18363	0,2993	1,6427003	0,5918	1,2060322	1,68885	4,1556348	3,31135	3,3014457	0,2845	0,7208006
Sint-112	2,38905	4,9917468	1,3345	3,3742099	3,6762	11,269773	0,5208	2,8583974	1,1796	2,4039128	3,375	8,304626	3,29655	3,2866899	0,42715	1,0822143
Sint-113	0,5649	1,1803176	0,9539	2,4118837	3,61125	11,070662	0,45675	2,5068606	0,8401	1,712044	3,22065	7,9248278	3,19545	3,1858923	0,48785	1,236002
Sint-114	0,90265	1,8860217	0,7048	1,782048	2,4709	7,5748007	0,49495	2,7165203	0,8827	1,7988588	2,31285	5,6910679	3,1459	3,1364905	0,5464	1,3843425
Sint-115	1,8909	3,9508985	1,22795	3,104804	3,67965	11,280349	0,3922	2,1525796	1,0236	2,0859996	2,20005	5,4135089	3,22875	3,2190927	0,384	0,9728908
Sint-116	1,2534	2,6188884	1,39145	3,5182048	3,63855	11,154353	1,04325	5,7258507	2,02195	4,1205421	2,3515	5,7861713	3,2509	3,2411765	1,08995	2,7614644
Sint-117	1,9905	4,1590054	1,1518	2,912263	3,56425	10,926579	0,4657	2,5559824	0,7463	1,5208885	3,28205	8,0759104	3,33585	3,3258724	0,6341	1,6065366
Sint-118	2,70715	5,6563936	3,2439	8,2020228	3,5748	10,958921	3,2492	17,83315	3,3259	6,7778684	3,43845	8,460753	3,31795	3,3080259	3,32735	8,4300735
Sint-119	1,10865	2,3164438	0,84125	2,1270544	3,6436	11,169834	0,4101	2,2508233	1,4449	2,944569	1,6029	3,9441437	3,3145	3,3045862	0,45895	1,1627819
Sint-120	1,55665	3,2525073	1,2805	3,2376738	3,65285	11,198191	0,75125	4,1232162	1,5594	3,1779091	2,4625	6,0593012	3,2977	3,2878365	0,745	1,8875095
Sint-122	3,0148	6,299206	2,2014	5,5661188	3,6789	11,27805	1,533	8,413831	2,6341	5,3680456	2,62865	6,4681348	3,2407	3,231007	1,699	4,3045351
Sint-123	1,79695	3,7545967	1,15695	2,9252845	3,5176	10,783568	0,8035	4,409989	1,4725	3,0008152	2,48965	6,1261073	3,2433	3,2335992	0,6747	1,7093995
Sint-124	0,7578	1,5833682	1,2512	3,1635904	3,66395	11,232219	0,632	3,4687157	1,0581	2,1563073	2,31385	5,6935285	3,2419	3,2322034	0,668	1,6924246
Sint-125	2,70595	5,6538863	1,2413	3,1385588	3,62985	11,127682	0,65165	3,5765642	1,2465	2,5402486	1,95345	4,8067175	3,25645	3,2467099	0,77725	1,9692171
Sint-126	1,7731	3,7047639	1,4691	3,7145386	3,5918	11,011036	0,7792	4,2766191	1,1701	2,3845527	2,01155	4,9496801	3,37125	3,3611665	0,67015	1,6978718
Sint-127	0,87285	1,8237568	1,01735	2,5723135	3,5674	10,936235	1,03585	5,685236	0,9164	1,8675362	2,14785	5,285064	3,36795	3,3578764	0,6288	1,5931087
Sint-128	1,40295	2,9313623	0,9608	2,42933	3,61735	11,089362	0,62895	3,4519759	2,05495	4,1877929	2,54075	6,2518455	3,3251	3,3151545	0,539	1,3655941
Sint-129	0,96145	2,0088801	1,4212	3,593426	3,6698	11,250153	0,6924	3,8002195	1,89235	3,8564296	1,72275	4,2390502	3,31395	3,3040379	0,7381	1,8700279
Sint-130	1,03285	2,1580652	0,9244	2,3372946	3,65825	11,214746	0,80065	4,3943469	2,9855	6,0841655	3,3133	8,1528051	3,2394	3,2297109	0,7452	1,8880162
Sint-132	3,49525	7,3030715	1,62425	4,1068268	3,63445	11,141784	0,31175	1,7110318	0,7312	1,4901162	2,8605	7,0386319	3,27455	3,2647557	1,229	3,1137573
Sint-133	0,96	2,0058504	0,6311	1,5957016	3,50045	10,730993	0,442	2,4259056	1,0393	2,1179947	2,5283	6,2212106	3,23575	3,2260718	0,4232	1,0722067
Sint-136	0,6079	1,270163	0,5334	1,3486726	2,46375	7,5528817	0,4161	2,2837541	0,8752	1,7835745	1,363	3,3538386	3,1432	3,1337986	0,4681	1,185964
Sint-137	1,29455	2,7048684	0,8825	2,2313527	3,4807	10,670448	0,4993	2,7403952	0,6455	1,3154677	1,8034	4,4375	3,35305	3,3430209	0,3712	0,9404611
Sint-138	1,2692	2,6519014	0,89145	2,2539823	3,58655	10,994942	0,45855	2,5167398	1,1201	2,2826574	1,5215	3,7438484	3,36845	3,3583749	0,9031	2,2880669
Sint-139	0,87465	1,8275178	0,67565	1,7083439	3,4229	10,493256	0,4322	2,3721186	0,7374	1,5027512	1,0847	2,6690453	3,3199	3,3099701	0,7075	1,7925006
Sint-140	1,57785	3,2968032	1,17245	2,9644753	3,6768	11,271613	0,5607	3,0773875	2,1172	4,3146525	1,94145	4,77719	3,33145	3,3214855	0,6021	1,5254624
Sint-142	1,46875	3,0688466	0,78585	1,9869785	3,6766	11,270999	0,3764	2,0658617	0,6001	1,2229468	1,3755	3,3845965	3,2822	3,2723829	0,5229	1,3248036
Sint-A36	2,4876	5,1976598	1,09595	2,7710493	1,1661	3,5748007	0,90855	4,9865532	2,1415	4,3641736	0,97835	2,4073573	2,2655	2,2587238	1,03885	2,631999
Sint-A40	1,4295	2,9868366	1,4471	3,6589128	3,6874	11,304108	0,6667	3,6591658	1,10075	2,243224	1,9499	4,7979823	3,27875	3,2689432	0,8263	2,0934887
Sint-B53	1,24755	2,6066653	1,0992	2,7792668	3,5971	11,027284	0,7128	3,9121844	1,7169	3,4988792	2,1178	5,211122	3,29035	3,2805085	0,8655	2,1928047
Sint-C55	0,92845	1,939929	0,803	2,0303413	1,6388	5,0239117	0,63875	3,5057629	1,38025	2,8128184	0,7343	1,8068406	3,08885	3,0796112	0,8592	2,1768432

Sint-C56	0,99375	2,0763686	1,3438	3,3977244	3,52595	10,809166	1,27925	7,0211306	2,5443	5,1850418	1,24125	3,0542569	3,33735	3,3273679	1,29175	3,2727388
Sint-C64	0,7602	1,5883828	1,23655	3,1265487	3,56165	10,918608	0,8476	4,6520307	1,56795	3,1953332	1,0681	2,6281988	3,27605	3,2662512	0,9794	2,4813783
Sint-D80	1,6333	3,4126619	1,27605	3,2264223	3,33885	10,235592	0,96415	5,2917124	1,8134	3,695537	1,50085	3,6930364	3,2386	3,2289133	0,8143	2,0630859
Sint-E89	2,2773	4,7582532	3,11635	7,8795196	3,5654	10,930104	3,0414	16,692645	3,21905	6,5601182	2,2546	5,5477362	3,2397	3,23001	2,97075	7,5266025
Sint-E91	1,0159	2,1226494	0,7312	1,848799	1,7401	5,3344574	0,85475	4,6912733	1,25535	2,5582841	0,3209	0,7896161	2,69125	2,6832004	0,6654	1,6858373
Sint-F109	1,9281	4,0286252	0,9618	2,4318584	3,6408	11,161251	0,66255	3,6363886	1,5108	3,0788669	1,3118	3,2278543	3,1946	3,1850449	0,83075	2,1047631
Sint-F111	1,7471	3,6504388	2,74395	6,9379267	3,6718	11,256284	2,64695	14,527717	3,05945	6,2348686	2,1349	5,2531988	3,24525	3,2355434	2,9297	7,4225994
Sint-G127	0,9663	2,0190138	1,29085	3,2638432	3,6747	11,265175	0,63385	3,4788694	1,11955	2,2815366	1,3424	3,3031496	3,22865	3,218993	0,6093	1,5437041
Sint-H140	1,3455	2,8113247	1,54015	3,8941846	3,64695	11,180104	0,969	5,3183315	2,319	4,7259018	1,43495	3,5308809	3,24305	3,23335	1,22995	3,1161642
Assi-05	1,02005	2,1313205	0,7557	1,9107459	0,9572	2,9343961	0,76335	4,1896268	1,37315	2,7983493	0,6163	1,5164862	2,7297	2,7215354	0,6289	1,593362
Assi-16	0,934	1,9515253	1,0057	2,5428571	1,96375	6,0200797	1,0741	5,8951701	2,3774	4,8449154	3,00005	7,3820128	2,62045	2,6126122	0,6453	1,6349126
Assi-26	1,45335	3,0366695	1,46625	3,7073325	3,64955	11,188075	0,791	4,3413831	1,2544	2,5563481	2,17635	5,3551919	3,2895	3,279661	0,6209	1,5730935
Assi-30	1,01075	2,1118888	1,01145	2,5573957	3,62535	11,113887	0,6148	3,3743139	1,0589	2,1579376	1,9435	4,7822343	3,2866	3,2767697	0,4288	1,0863947
Assi-50	0,77355	1,6162766	0,41935	1,0603034	0,8098	2,4825261	0,76935	4,2225576	1,851	3,7721622	0,5275	1,2979823	1,2488	1,2450648	0,6882	1,7436027
Assi-51	0,58755	1,2276431	0,5218	1,3193426	0,5672	1,7388105	0,4842	2,6575192	0,7818	1,5932342	0,4194	1,0319882	2,9456	2,9367896	0,3321	0,8413985
Assi-52	1,1536	2,4103636	1,5014	3,7962073	3,73995	11,465205	0,7334	4,025247	1,5608	3,1807622	1,2746	3,1363189	3,225	3,2153539	0,6646	1,6838105
Assi-53	0,89165	1,863038	0,4929	1,2462705	0,60815	1,864347	0,4905	2,6920966	0,7369	1,5017322	0,3877	0,9539862	1,58295	1,5782154	0,4707	1,1925513
Assi-54	1,35055	2,8218763	1,0549	2,6672566	3,57065	10,946199	0,7398	4,0603732	1,305	2,6594661	1,3252	3,2608268	3,33615	3,3261715	0,5335	1,3516595
Assi-55	0,8436	1,762641	1,1718	2,9628319	0,86345	2,6469957	0,9514	5,2217344	1,7952	3,6584471	0,5411	1,3314469	0,9054	0,9026919	0,6675	1,6911578
Assi-56	0,9076	1,8963644	0,63005	1,5930468	0,7147	2,1909871	0,819	4,4950604	1,29775	2,6446913	0,50645	1,246186	0,86515	0,8625623	0,91935	2,3292374
Assi-57	0,94495	1,9744045	1,2206	3,08622	0,77615	2,3793685	0,8487	4,6580681	1,90585	3,8839413	0,7554	1,8587598	1,06895	1,0657527	0,74215	1,8802888
Assi-58	0,498	1,0405349	0,7899	1,9972187	3,63865	11,15466	0,4827	2,6492865	0,503	1,0250662	1,9181	4,7197343	3,1183	3,1089731	0,3548	0,8989106
Assi-59	1,0319	2,1560802	1,0818	2,7352718	3,68795	11,305794	0,59855	3,2851262	1,0341	2,1073976	1,6938	4,167815	3,2729	3,2631107	0,5244	1,328604
Assi-60	0,99105	2,0707271	2,0536	5,1924147	3,6867	11,301962	0,9123	5,007135	1,12005	2,2825555	1,4398	3,542815	3,23785	3,2281655	2,722	6,896377
Assi-62	1,0418	2,1767656	1,0881	2,751201	3,603	11,045371	0,8004	4,3929748	1,0702	2,180966	1,8468	4,5442913	3,24795	3,2382353	0,6092	1,5434507
Assi-63	1,0212	2,1337234	1,2215	3,0884956	1,2134	3,7198038	1,06895	5,8669045	2,2825	4,6515182	0,6183	1,5214075	1,1562	1,1527418	0,858	2,1738029
Assi-64	0,5948	1,2427915	0,45225	1,1434893	0,62175	1,9060392	0,87035	4,7768935	0,7291	1,4858366	0,45905	1,1295522	2,2064	2,1998006	0,4013	1,0167216
Assi-65	2,30515	4,8164438	0,9647	2,4391909	3,5575	10,905886	0,5473	3,0038419	0,7461	1,5204809	1,4221	3,4992618	3,30965	3,2997507	1,5133	3,8340512
Assi-66	0,66885	1,3975136	0,61445	1,553603	0,48905	1,4992336	0,6117	3,3572997	0,9701	1,9769717	0,5291	1,3019193	1,2529	1,2491525	0,4763	1,2067393
Assi-67	2,1612	4,5156707	0,79645	2,01378	3,6076	11,059473	0,51775	2,8416575	1,0858	2,2127573	2,8114	6,917815	3,1786	3,1690927	0,5011	1,2695718
Assi-68	1,94135	4,0563101	1,09445	2,7672566	3,72285	11,412784	0,5972	3,2777168	1,2633	2,5744854	1,68265	4,1403789	3,2752	3,2654038	1,1057	2,8013681
Assi-69	0,7	1,4625992	0,6242	1,5782554	2,19455	6,7276211	0,4426	2,4291987	0,7251	1,4776849	0,82105	2,0203002	3,15685	3,1474078	0,4295	1,0881682
Assi-70	0,8351	1,7448809	0,6307	1,5946903	0,38845	1,1908338	0,5534	3,0373216	0,5407	1,1018953	0,9215	2,2674705	0,7933	0,7909272	0,371	0,9399544
Assi-72	1,19015	2,4867321	0,5006	1,2657396	1,51055	4,630748	0,4931	2,7063666	0,77015	1,5694926	1,889	4,6481299	2,09675	2,0904786	0,4118	1,043324
Assi-73	0,701	1,4646887	0,6913	1,747914	3,5745	10,958001	0,4391	2,409989	0,5079	1,035052	1,2714	3,1284449	3,36875	3,358674	0,3397	0,8606537
Assi-74	0,89985	1,8801713	0,84895	2,1465234	3,2165	9,860515	0,4162	2,284303	0,5466	1,1139189	0,8674	2,1343504	3,28155	3,2717348	0,3383	0,8571067
Assi-75	1,2033	2,5142081	1,31185	3,3169406	3,694	11,324341	0,8035	4,409989	1,7425	3,5510495	1,1459	2,8196358	3,3535	3,3434696	0,5742	1,4547758
Assi-76	1,0414	2,1759298	0,4041	1,0217446	2,0332	6,2329859	0,45405	2,4920417	0,5765	1,1748523	0,4299	1,0578248	2,7956	2,7872383	0,4495	1,1388396
Assi-77	0,62575	1,3074593	0,77735	1,9654867	3,653	11,198651	0,61445	3,372393	0,8452	1,7224373	2,07765	5,1123278	3,31105	3,3011466	0,4547	1,1520142
Assi-78	2,49305	5,2090472	0,99225	2,5088496	3,5928	11,014102	0,7198	3,9506037	1,4317	2,9176686	1,08565	2,6713829	3,257	3,2472582	0,7608	1,9275399
Assi-79	1,2212	2,5516089	0,71835	1,8163085	3,63125	11,131974	0,43205	2,3712953	0,7439	1,5159976	1,3331	3,2802657	3,3023	3,2924227	0,4252	1,0772739
Assi-80	0,70705	1,4773297	1,6908	4,2750948	3,58915	11,002912	1,0227	5,6130626	0,9649	1,9663746	0,98185	2,4159695	3,3403	3,3303091	0,8601	2,1791234
Assi-81	0,7124	1,4885081	1,0264	2,595196	3,6377	11,151747	0,983	5,3951701	1,0676	2,1756674	1,83545	4,5163632	3,34565	3,3356431	0,99195	2,5131746
Assi-82	2,307	4,8203092	1,47355	3,7257901	3,5923	11,012569	0,54815	3,0085071	2,3503	4,7896882	1,22535	3,0151329	3,2623	3,2525424	1,0345	2,620978
Assi-83	1,2661	2,6454242	0,6289	1,5901391	3,5891	11,002759	0,352	1,9319429	0,9352	1,9058488	1,0891	2,679872	3,2657	3,2559322	0,4553	1,1535343
Assi-D70	2,67355	5,5861889	0,851	2,1517067	1,46985	4,5059779	0,2962	1,6256861	1,1614	2,3668229	0,8713	2,1439469	2,90435	2,895663	0,5315	1,3465923
Assi-E92	1,68	3,5102382	2,53615	6,4125158	3,4451	10,561312	1,192	6,5422613	3,0295	6,1738333	1,14685	2,8219734	3,2928	3,2829511	0,7585	1,9217127
Assi-G122	1,11255	2,3245926	0,6938	1,7542351	1,24055	3,8030349	0,3091	1,6964874	1,32395	2,6980844	1,02525	2,5227608	2,56515	2,5574776	0,7517	1,9044844
Assi-G123	1,67355	3,4967614	1,04125	2,6327434	1,1986	3,6744329	0,4522	2,481888	1,30605	2,6616059	0,61735	1,5190699	2,00005	1,9940678	0,66925	1,6955916
Assi-G124	1,18025	2,4660468	0,76175	1,926043	1,63015	4,9973942	0,6217	3,4121844	1,38265	2,8177094	0,50795	1,249877	2,5299	2,522333	0,9293	2,3544464

Assi-H132	1,13785	2,3774551	1,64345	4,1553729	1,3042	3,9981606	1,2113	6,6481888	3,02015	6,1547789	0,803	1,9758858	1,75555	1,7502991	1,1388	2,8852293
Assi-H141	1,8823	3,9329294	1,16405	2,9432364	2,1199	6,4987738	0,2791	1,5318332	1,25835	2,5643978	3,19765	7,8682333	3,2073	3,1977069	0,64075	1,6233848
Assi-A21	1,34575	2,8118471	1,01525	2,5670038	0,9206	2,822195	0,556	3,0515917	1,75105	3,5684736	0,5707	1,4042815	1,42875	1,4244766	1,1055	2,8008614
Assi-G123	2,0602	4,3046385	1,0847	2,7426043	0,8639	2,6483752	0,2184	1,1986828	1,2712	2,5905849	0,58055	1,4285187	1,70525	1,7001496	0,6451	1,6344059
Assi-G124	1,86875	3,9046176	0,82785	2,0931732	1,4752	4,5223789	0,3238	1,7771679	1,1771	2,398818	0,2309	0,5681594	2,20505	2,1984546	0,9423	2,3873828
Assi-H132	1,63715	3,4207062	1,96415	4,9662453	0,63135	1,935469	1,09325	6,0002744	2,68445	5,4706542	0,7275	1,7901083	1,2254	1,2217348	0,9512	2,4099316
Assi-141	0,64025	1,337756	0,17835	0,4509482	0,1451	0,4448191	0,4348	2,3863886	1,39705	2,8470552	0,076	0,1870079	3,16125	3,1517946	0,7845	1,9875855
Assi-A4	2,303	4,8119515	1,16185	2,9376738	1,5488	4,7480074	0,601	3,298573	2,2726	4,631343	0,73205	1,8013041	2,7841	2,7757727	1,02115	2,5871548
Assi-A15	1,70905	3,5709361	1,4813	3,7453856	1,8777	5,7562845	0,9362	5,1383095	2,18915	4,4612798	0,84605	2,0818159	3,23535	3,225673	1,1116	2,8163162
B47	1,0929	2,2835353	0,27515	0,6957016	0,4999	1,5324954	0,22145	1,2154226	0,331	0,6745466	0,3045	0,7492618	0,6967	0,6946162	0,3636	0,921206
B48	2,20145	4,5997702	1,2496	3,1595449	2,9392	9,0104231	0,7591	4,1663008	2,1353	4,3515386	0,7343	1,8068406	3,26835	3,2585743	1,2056	3,0544718
B50	1,52455	3,1854367	0,6597	1,6680152	0,83475	2,5590129	0,3691	2,0257958	0,9502	1,9364174	0,45505	1,1197096	2,4088	2,4015952	0,4478	1,1345326
B51	1,53925	3,2161513	1,2427	3,1420986	0,57955	1,7766708	0,3037	1,6668496	1,745	3,5561443	0,69405	1,7078002	1,06425	1,0610668	0,5739	1,4540157
B54	1,89485	3,9591517	0,8146	2,0596713	1,39035	4,2622624	0,1974	1,0834248	1,5472	3,1530467	0,8292	2,0403543	2,5199	2,5123629	0,6951	1,7610844
C65	1,5211	3,1782282	0,6476	1,637421	0,46475	1,4247394	0,2212	1,2140505	1,1145	2,2712452	0,6689	1,6459154	1,35345	1,3494018	0,6743	1,7083861
D71	1,44715	3,023715	0,80065	2,0243995	0,61545	1,8867259	0,3342	1,8342481	1,4369	2,9282657	0,8828	2,1722441	1,52655	1,521984	0,8181	2,0727135
D83	1,0946	2,2870873	0,8281	2,0938053	1,1274	3,4561619	0,3602	1,9769484	1,2292	2,5049929	0,8541	2,101624	2,8204	2,8119641	0,60365	1,5293894
D86	1,39675	2,9184079	0,78135	1,9756005	1,29165	3,9596873	0,2447	1,3430296	0,93655	1,9086	0,7449	1,8329232	2,0173	2,0112662	0,5866	1,486192
F113	1,2854	2,6857501	0,5142	1,3001264	0,4875	1,4944819	0,1312	0,7200878	0,7388	1,5056042	0,54135	1,332062	0,7508	0,7485543	0,58015	1,4698505
G126	1,61305	3,370351	0,9892	2,5011378	3,50125	10,733446	0,1428	0,7837541	0,9439	1,9235786	1,5449	3,8014272	3,3171	3,3071785	0,6174	1,564226
H139	1,0515	2,197033	0,5519	1,3954488	0,35085	1,0755671	0,3883	2,1311745	1,0051	2,0482983	0,6009	1,4785925	1,9766	1,9706879	0,5527	1,400304
H142	1,1764	2,4580025	0,8209	2,0756005	2,8187	8,6410178	0,2012	1,104281	1,00735	2,0528836	0,5642	1,3882874	3,21115	3,2015454	0,7546	1,9118318
H146	1,6222	3,3894693	0,646	1,6333755	0,607	1,8608216	0,3224	1,7694841	1,0618	2,1638476	0,40925	1,0070128	1,33295	1,3289631	0,643	1,6290854
H147	1,0147	2,1201421	0,62805	1,5879899	0,97065	2,9756284	0,3913	2,14764	0,8935	1,8208681	0,5862	1,4424213	2,67605	2,6680459	0,3914	0,9916392
H148	0,90245	1,8856038	0,6634	1,6773704	1,3228	4,0551809	0,3717	2,0400659	1,1	2,2416955	0,55875	1,374877	1,6784	1,6733799	0,7372	1,8677477
H149	1,4107	2,9475554	1,26645	3,2021492	2,16615	6,6405579	0,5641	3,0960483	1,88855	3,8486856	0,8034	1,9768701	3,31765	3,3077268	0,9081	2,3007347
H150	1,00585	2,1016506	0,461	1,1656131	1,91925	5,8836603	0,3491	1,9160263	0,6518	1,3283065	1,01965	2,5089813	3,2421	3,2324028	0,39675	1,0051938
H153	0,9876	2,0635186	0,71	1,795196	0,706	2,1643164	0,4235	2,3243688	0,8501	1,7324231	0,5276	1,2982283	1,6967	1,6916251	0,5926	1,5013935
Bab-1	0,5992	1,251985	0,1211	0,3061947	-0,0325	-0,099632	-0,049	-0,268935	0,0185	0,0377012	0,32005	0,7875246	1,2752	1,2713858	0,31785	0,8052952
Bab-4	0,0656	0,1370664	0,04885	0,1235145	-0,0986	-0,302269	-0,07385	-0,405324	0,1983	0,4041166	0,1906	0,4689961	0,3775	0,3763709	0,49815	1,2620978
Bab-8	0,3449	0,7206435	0,23665	0,5983565	-0,00295	-0,009044	0,0479	0,2628979	0,2627	0,5353577	0,3894	0,9581693	0,4032	0,401994	0,4283	1,0851279
Bab-11	0,6887	1,4389887	0,27535	0,6962073	0,08745	0,2680871	0,0756	0,4149286	0,3	0,6113715	0,4798	1,1806102	0,3803	0,3791625	0,4573	1,1586015
Bab-21	-0,07635	-0,159528	0,0065	0,0164349	0,04295	0,1316677	0,01695	0,0930296	-0,2106	-0,429183	0,2319	0,5706201	0,4104	0,4091725	0,26065	0,660375
Bab-24	0,0761	0,1590054	0,2013	0,508976	1,64905	5,0553342	0,0372	0,2041712	0,01245	0,0253719	0,2198	0,5408465	0,8357	0,8332004	0,3248	0,8229035
Bab-26	0,1896	0,3961555	0,0476	0,120354	2,76495	8,4762416	0,05845	0,3208013	0,0694	0,1414306	0,4588	1,128937	1,14385	1,1404287	0,2812	0,7124398
Ehrl-23	0,09695	0,20257	0,1874	0,4738306	0,09495	0,2910791	0,15	0,8232711	0,1599	0,325861	0,2557	0,6291831	1,22755	1,2238784	0,46	1,1654421
Ehrl-29	-0,17245	-0,360322	0,01835	0,046397	-0,1705	-0,522685	0,093	0,5104281	0,11	0,2241696	0,12705	0,312623	0,6459	0,6439681	0,3925	0,9944261
Ehrl-30	-0,0947	-0,197869	0,14995	0,3791403	0,04125	0,1264562	0,1076	0,5905598	0,0872	0,1777053	0,3467	0,8531004	0,5488	0,5471585	0,4082	1,0342032
Ehrl-34	0,27505	0,574697	0,7283	1,8414665	0,3015	0,9242796	0,5477	3,0060373	0,0952	0,1940086	0,3263	0,8029035	0,8933	0,8906281	0,7277	1,8436787
Ehrl-36	0,0245	0,051191	0,09455	0,2390645	-0,00455	-0,013948	0,3079	1,6899012	0,0562	0,1145303	0,2095	0,515502	2,0697	2,0635095	0,7475	1,8938434
Ehrl-37	0,17375	0,363038	0,16115	0,4074589	0,21485	0,658645	0,1167	0,6405049	0,201	0,4096189	0,3421	0,8417815	0,767	0,7647059	0,3175	0,8044084
61/17	0,0256	0,0534893	0,2235	0,5651075	0,20335	0,6233906	0,2755	1,5120746	0,1883	0,3837375	0,5409	1,3309547	1,26575	1,2619641	0,7391	1,8725614
5 A	-0,0301	-0,062892	-0,0036	-0,009102	-0,0111	-0,034028	0,08	0,4390779	0,6013	1,2253923	0,3437	0,8457185	0,579	0,5772682	0,3176	0,8046618
6 A	-0,0097	-0,020267	0,057	0,1441214	-0,0586	-0,179644	0,0406	0,2228321	0,26845	0,5470756	0,32635	0,8030266	0,8374	0,8348953	0,3893	0,9863187
8 A	-0,07175	-0,149916	0,0417	0,1054362	-0,0786	-0,240956	0,0801	0,4396268	0,6427	1,3097616	0,21995	0,5412156	0,6937	0,6916251	0,3781	0,9579427
9 A	0,049	0,1023819	0,0349	0,0882427	0,15545	0,4765481	0,0723	0,3968167	0,4657	0,9490524	0,2709	0,6665846	0,3126	0,311665	0,4492	1,1380796
14 A	-0,1393	-0,291057	0,03555	0,0898862	-0,10225	-0,313458	-0,0246	-0,135016	0,27045	0,5511514	0,18295	0,4501722	0,3506	0,3495513	0,36755	0,9312136
16 A	-0,0785	-0,16402	0,022	0,0556258	0,3	0,9196812	-0,02525	-0,138584	0,2193	0,4469126	0,19235	0,4733022	0,3654	0,3643071	0,3048	0,7722321
17 A	0,363	0,7584622	0,1633	0,4128951	0,11815	0,3622011	0,1436	0,7881449	0,227	0,4626044	0,2664	0,6555118	1,14175	1,138335	0,4078	1,0331898
19 A	0,09635	0,2013163	0,0576	0,1456384	0,0139	0,0426119	0,02435	0,1336443	0,5475	1,115753	0,31775	0,7818652	0,9325	0,9297109	0,3283	0,831771

20 A	0,187	0,3907229	0,11675	0,295196	0,11275	0,3456468	0,01785	0,0979693	1,4313	2,9168535	0,3241	0,7974902	0,532	0,5304088	0,3403	0,8621738
21 A	0,0427	0,0892186	0,0492	0,1243995	-0,0627	-0,192213	-0,0168	-0,092206	1,6768	3,4171592	0,25855	0,6361959	0,4439	0,4425723	0,2667	0,6757031
4 C	0,07605	0,158901	0,0605	0,1529709	0,0652	0,1998774	0,033	0,1811196	0,1999	0,4073772	0,4599	1,1316437	0,6837	0,681655	0,382	0,9678237
5 C	0,0153	0,0319682	0,11755	0,2972187	0,10695	0,3278663	0,0946	0,5192097	0,0553	0,1126961	0,26	0,6397638	0,7829	0,7805583	0,3926	0,9946795
6 C	-0,02725	-0,056937	0,10265	0,2595449	-0,0331	-0,101471	0,0933	0,5120746	0,2938	0,5987365	0,2117	0,5209154	0,8674	0,8648056	0,345	0,8740816
11 C	0,1004	0,2097785	0,12715	0,3214918	0,1944	0,5959534	0,1044	0,5729967	0,2032	0,4141023	0,36145	0,8893947	0,7502	0,7479561	0,4611	1,168229
16 C	0,11745	0,2454033	0,1385	0,3501896	0,2871	0,8801349	0,1669	0,9160263	1,5236	3,1049521	1,05805	2,6034695	2,9688	2,9599202	0,4505	1,1413732
17 C	0,164	0,3426661	0,11615	0,2936789	0,9764	2,9932557	0,0226	0,1240395	1,13375	2,3104748	0,934	2,2982283	0,81575	0,8133101	0,288	0,7296681
19 C	-0,03205	-0,066966	0,095	0,2402023	0,16685	0,511496	-0,0346	-0,189901	1,7108	3,4864479	0,5928	1,4586614	0,7477	0,7454636	0,3146	0,7970611
21 C	0,2254	0,470957	0,3869	0,9782554	0,2201	0,6747394	0,0495	0,2716795	0,4783	0,97473	0,99805	2,4558317	0,8786	0,8759721	0,7159	1,8137826
20 C	0,3087	0,6450063	0,12415	0,3139064	0,16565	0,5078173	-0,04165	-0,228595	0,091	0,1854494	0,4543	1,1178642	0,5664	0,5647059	0,5928	1,5019002
Neg-08	0,0222	0,0463853	0,0596	0,1506953	0,0657	0,2014102	-0,09665	-0,530461	-0,0091	-0,018545	0,3634	0,8941929	0,49785	0,4963609	0,35945	0,9106917
Neg-50	0,0525	0,1096949	0,156	0,3944374	0,12185	0,3735438	-0,0247	-0,135565	0,1801	0,3670267	0,26165	0,6438238	0,71105	0,7089232	0,7629	1,9328604
Neg-52	0,00695	0,0145215	0,059	0,1491783	0,0513	0,1572655	-0,03555	-0,195115	0,0967	0,1970654	0,2618	0,6441929	0,3833	0,3821535	0,5083	1,2878135
Neg-53	-0,0054	-0,011283	0,0488	0,1233881	0,0975	0,2988964	-0,0298	-0,163557	0,0885	0,1803546	0,35585	0,8756152	0,4397	0,4383848	0,4607	1,1672156
Neg-54	0,08305	0,173527	0,1831	0,4629583	0,04355	0,1335071	0,0339	0,1860593	0,2483	0,5060118	0,23705	0,5832923	0,5275	0,5259222	0,4907	1,2432227
Neg-56	0,45915	0,9593606	0,08885	0,2246523	0,1161	0,3559166	-0,0866	-0,475302	0,0536	0,1092317	0,3121	0,7679626	0,4137	0,4124626	0,28265	0,7161135
Neg-57	0,4136	0,8641872	0,16695	0,4221239	0,1735	0,5318823	-0,0344	-0,188804	0,1393	0,2838802	0,2815	0,6926673	0,4051	0,4038883	0,45005	1,1402331
Neg-60	0,1664	0,3476807	0,076	0,1921618	-0,00235	-0,007204	-0,073	-0,400659	0,0558	0,1137151	0,2815	0,6926673	0,4577	0,456331	0,3504	0,8877629
Neg-61	0,07265	0,1517969	0,1386	0,3504425	0,1086	0,3329246	-0,0194	-0,106476	0,0829	0,1689423	0,47175	1,1608022	0,788	0,7856431	0,4689	1,1879909
Neg-62	0,06315	0,1319473	0,09595	0,2426043	-0,0623	-0,190987	-0,09415	-0,51674	-0,0193	-0,039332	0,2702	0,6648622	0,3107	0,3097707	0,3602	0,9125918
Neg-64	-0,0068	-0,014208	0,11815	0,2987358	-0,0873	-0,267627	-0,05245	-0,28787	0,0117	0,0238435	0,17935	0,441314	0,3991	0,3979063	0,4967	1,2584241
Neg-65	-0,0196	-0,040953	0,0888	0,2245259	-0,0678	-0,207848	-0,0247	-0,135565	0,1094	0,2229468	0,1502	0,3695866	0,1979	0,1973081	0,5224	1,3235369
Neg-66	0,2184	0,456331	0,1877	0,4745891	0,1017	0,3117719	0,0496	0,2722283	0,1829	0,3727328	0,28955	0,7124754	0,5139	0,5123629	0,4936	1,2505701
Neg-79	0,08315	0,1737359	0,1588	0,4015171	0,12715	0,3897915	0,0031	0,0170143	0,1012	0,206236	0,33425	0,8224656	0,653	0,6510469	0,4233	1,0724601
Neg-81	0,158	0,3301295	0,2657	0,6718078	0,01635	0,0501226	-0,06435	-0,353183	0,1497	0,3050744	0,26895	0,6617864	0,3829	0,3817547	0,3759	0,9523689
Neg-82	-0,00195	-0,004074	0,0512	0,1294564	-0,05775	-0,177039	-0,10015	-0,549671	-0,0288	-0,058692	0,2528	0,6220472	0,3943	0,3931206	0,31905	0,8083354
Neg-83	-0,0117	-0,024446	-0,01895	-0,047914	-0,10895	-0,333998	-0,05915	-0,324643	-0,08415	-0,17149	0,23845	0,5867372	0,3532	0,3521436	0,3729	0,9447682
Neg-84	-0,04475	-0,093502	0,03495	0,0883692	-0,07355	-0,225475	-0,0258	-0,141603	-0,08735	-0,178011	0,29405	0,7235482	0,32985	0,3288634	0,4224	1,0701799
Neg-108	0,0095	0,0198496	0,0716	0,1810367	0,04155	0,1273758	-0,0137	-0,075192	0,0269	0,0548196	0,268	0,6594488	0,7693	0,766999	0,7017	1,7778059
Neg-124	0,13705	0,286356	0,1708	0,4318584	0,0661	0,2026364	0,0616	0,33809	0,1639	0,3340126	0,30885	0,7599656	0,6443	0,6423729	0,54705	1,3859894
Neg-131	0,35715	0,746239	0,36365	0,919469	0,0671	0,205702	0,0408	0,2239297	0,1545	0,3148563	0,2741	0,6744587	0,635	0,6331007	0,5089	1,2893337
Neg-151	0,3456	0,7221061	0,0766	0,1936789	0,10185	0,3122318	0,06195	0,340011	0,0494	0,1006725	0,2971	0,7310531	0,6838	0,6817547	0,6333	1,6045098
Neg-152	0,06585	0,1375888	0,1966	0,4970923	-0,0187	-0,057327	0,0792	0,4346872	0,3024	0,6162625	0,234	0,5757874	0,6811	0,6790628	0,8629	2,1862174
Neg-153	0,16915	0,3534267	0,25165	0,6362832	0,08745	0,2680871	-0,0042	-0,023052	0,1	0,2037905	0,15865	0,3903789	1,499	1,4945165	0,5479	1,3881429
Neg-156	0,04435	0,0926661	0,2255	0,5701643	0,12265	0,3759963	0,04425	0,242865	-0,0383	-0,078052	0,2562	0,6304134	0,6355	0,6335992	0,4272	1,082341
Neg-157	0,22915	0,4787923	0,07405	0,1872314	0,21785	0,6678418	-0,01005	-0,055159	-0,0228	-0,046464	0,1759	0,4328248	0,6372	0,6352941	0,4691	1,1884976
Neg-172	0,29095	0,6079189	0,35935	0,9085967	0,20335	0,6233906	0,0827	0,4538968	0,2685	0,5471775	0,35365	0,8702018	1,2764	1,2725823	0,2631	0,6665822
Neg-159	-0,0511	-0,10677	-0,0006	-0,001517	-0,03805	-0,116646	-0,0985	-0,540615	-0,07265	-0,148054	0,21295	0,5239911	0,3153	0,3143569	0,16385	0,4151254
Neg-160	0,32665	0,6825115	0,17505	0,4426043	0,1131	0,3467198	0,0744	0,4083425	0,1802	0,3672305	0,2812	0,6919291	0,7507	0,7484546	0,31955	0,8096022
Neg-161	0,1399	0,2923109	0,0555	0,1403287	0,08135	0,2493869	-0,0154	-0,084523	0,0285	0,0580803	0,2603	0,640502	0,9417	0,9388833	0,2683	0,6797568
Neg-166	0,15605	0,3260552	0,26835	0,6785082	0,1701	0,5214592	0,05945	0,3262898	0,1183	0,2410842	0,29795	0,7331447	2,3702	2,3631107	0,058	0,146947
Neg-167	0,1237	0,2584622	0,21415	0,5414665	0,1959	0,6005518	0,2258	1,2392975	0,2108	0,4295904	0,26315	0,6475148	1,22215	1,2184945	0,35065	0,8883963
Neg-171	0,21315	0,4453615	0,13995	0,3538559	0,20965	0,6427039	-0,0022	-0,012075	0,0657	0,1338904	0,40355	0,9929872	0,648	0,6460618	0,4615	1,1692425
Neg-158	0,2188	0,4571667	0,1598	0,4040455	0,3	0,9196812	-0,0453	-0,248628	0,155	0,3158753	0,29455	0,7247785	0,5891	0,587338	0,4262	1,0798074
