# Supplementary material for: Immunogenic mapping of rDyn-1 and rKDDR-plus proteins and selection of oligopeptides by immunoblotting for the diagnosis of Leishmania infantum-infected dogs
Source: PLoS Negl Trop Dis. 2023 Aug 4;17(8):e0011535. doi: 10.1371/journal.pntd.0011535 (PMC10442149; doi:10.1371/journal.pntd.0011535)
Supplement: S2 File — Reports of studies of diagnostic accuracy. (PDF) [file pntd.0011535.s005.pdf]

Section & Topic	No	Item	Reported on page #
**TITLE OR ABSTRACT**			
	1	Identification as a study of diagnostic accuracy using at least one measure of accuracy (such as sensitivity, specificity, predictive values, or AUC)	Page 2
**ABSTRACT**			
	2	Structured summary of study design, methods, results, and conclusions (for specific guidance, see STARD for Abstracts)	Page 2
**INTRODUCTION**			
	3	Scientific and clinical background, including the intended use and clinical role of the index test	Page 4 and 5
	4	Study objectives and hypotheses	Page 6
**METHODS**			
Study design	5	Whether data collection was planned before the index test and reference standard were performed (prospective study) or after (retrospective study)	Page 7, 8 and 9
Participants	6	Eligibility criteria	Page 7, 8 and 9
	7	On what basis potentially eligible participants were identified (such as symptoms, results from previous tests, inclusion in registry)	Page 7, 8 and 9
	8	Where and when potentially eligible participants were identified (setting, location and dates)	Page 7, 8 and 9
	9	Whether participants formed a consecutive, random or convenience series	Page 7, 8 and 9
Test methods	10a	Index test, in sufficient detail to allow replication	Page 15 and 16
	10b	Reference standard, in sufficient detail to allow replication	Page 15 and 16
	11	Rationale for choosing the reference standard (if alternatives exist)	
	12a	Definition of and rationale for test positivity cut-offs or result categories of the index test, distinguishing pre-specified from exploratory	Page 16 and 17
	12b	Definition of and rationale for test positivity cut-offs or result categories of the reference standard, distinguishing pre-specified from exploratory	
	13a	Whether clinical information and reference standard results were available to the performers/readers of the index test	
	13b	Whether clinical information and index test results were available to the assessors of the reference standard	
Analysis	14	Methods for estimating or comparing measures of diagnostic accuracy	Page 16 and 17
	15	How indeterminate index test or reference standard results were handled	
	16	How missing data on the index test and reference standard were handled	
	17	Any analyses of variability in diagnostic accuracy, distinguishing pre-specified from exploratory	
	18	Intended sample size and how it was determined	
**RESULTS**			
Participants	19	Flow of participants, using a diagram	
	20	Baseline demographic and clinical characteristics of participants	
	21a	Distribution of severity of disease in those with the target condition	
	21b	Distribution of alternative diagnoses in those without the target condition	
	22	Time interval and any clinical interventions between index test and reference standard	
Test results	23	Cross tabulation of the index test results (or their distribution) by the results of the reference standard	Table 1 and 2<br>Pages 27 and 29
	24	Estimates of diagnostic accuracy and their precision (such as 95% confidence intervals)	Table 1 and 2<br>Pages 27 and 29
	25	Any adverse events from performing the index test or the reference standard	
**DISCUSSION**			
	26	Study limitations, including sources of potential bias, statistical uncertainty, and generalisability	Page 33 and 34
	27	Implications for practice, including the intended use and clinical role of the index test	Page 33 and 34
**OTHER INFORMATION**			

CC BY SA

	**28**	Registration number and name of registry	
	**29**	Where the full study protocol can be accessed	
	**30**	Sources of funding and other support; role of funders	
